# Development of a bioavailability-based acute effects assessment method for nickel

**DOI:** 10.1093/etojnl/vgae071

**Published:** 2025-01-06

**Authors:** Charlotte Nys, Elizabeth Middleton, Emily Garman, Chris Schlekat, Patrick Van Sprang, Karel De Schamphelaere

**Affiliations:** ARCHE Consulting, Ghent, Belgium; NiPERA, Durham, NC, United States; NiPERA, Durham, NC, United States; NiPERA, Durham, NC, United States; ARCHE Consulting, Ghent, Belgium; ARCHE Consulting, Ghent, Belgium; GhEnToxLab, Ghent University, Ghent, Belgium

**Keywords:** metal toxicity, freshwater toxicology, biotic ligand model, risk assessment

## Abstract

This study aimed to develop a bioavailability-based effects assessment method for nickel (Ni) to derive acute freshwater environmental thresholds in Europe. The authors established a reliable acute freshwater Ni ecotoxicity database covering 63 different freshwater species, and the existing acute Ni bioavailability models for invertebrates were revised. A single average invertebrate bioavailability model was proposed, in which the protective effects of Ca^2+^ and Mg^2+^ on Ni^2+^ toxicity were integrated as a single-site competition effect at the Ni biotic ligand. The biotic ligand stability constants for Ca^2+^ and Mg^2+^ (log K_CaBL_ = 3.80 and log K_MgBL_ = 3.32) were derived by averaging these parameters from three existing cladoceran models. A pH extension was also integrated into the average invertebrate bioavailability model to reflect the increase in free Ni^2+^ toxicity observed greater than pH 8.0. The proposed invertebrate model has further been validated using an extensive dataset of acute toxicity data covering 15 different invertebrate species. Evaluating the extrapolation of the invertebrate model to plant species revealed significant uncertainty about the applicability of the acute Ni bioavailability models for plants. The newly developed acute invertebrate model was used alongside the existing acute fish and algae bioavailability models to support an acute bioavailability normalization approach for Ni. By combining these bioavailability models with the acute toxicity dataset for Ni, a normalized species sensitivity distribution approach is proposed to derive site-specific acute environmental thresholds, expressed by the HC5_L(E)C50_ (i.e., dissolved Ni concentration resulting in at least 50% effect for 5% of the species). The applicability ranges of the acute Ni bioavailability normalization approach are estimated to be valid for approximately 70% of European freshwaters. The proposed approach serves as a basis to incorporate bioavailability into the compliance evaluation relative to acute environmental threshold values for Ni in Europe.

## Introduction

The European Union (EU) Water Framework Directive (WFD) aims to protect European freshwater systems by ensuring good ecological and chemical status (European Commission [EC], 2008a). Under the WFD, nickel (Ni) is classified as a priority substance (EC, 2000), meaning that EU-wide environmental quality standards (EQS) are in place for Ni and all EU member states are subject to mandatory monitoring and compliance with the Ni EQS. Furthermore, the EU Registration, Evaluation, Authorisation and Restriction of Chemicals (REACH; EC, 2006) regulation aims to improve the protection of human health and the environment against exposure to potentially harmful substances (e.g., Ni). Both EU regulations consider two types of environmental threshold levels for the aquatic compartment (EC, 2018; European Chemicals Agency [ECHA], 2008a). The first is an environmental threshold intended to protect freshwater ecosystems against long-term effects, that is, the annual average–environmental quality standard (AA-EQS) under the WFD and the predicted no-effect concentration (PNEC) under REACH. A second environmental threshold aims to protect aquatic ecosystems against short-term effects occurring during peak exposures resulting from, for example, effluent discharges, calamities, or stormwater releases. It is assumed that if the peak exposure is limited in time, populations can tolerate higher levels than when the exposure is long-lasting. Mebane (2022) reported that recovery of fish and insect communities occurred faster when communities were affected by (acute) pulse exposures compared with (chronic) continuous exposure concentrations of aquatic contaminants. Within the WFD, peak exposures are targeted with the maximum allowable concentration-environmental quality standard (MAC-EQS), whereas under REACH, these are regulated using the PNEC for intermittent releases (PNEC_intermittent_). Both the MAC-EQS and PNEC_intermittent_ are derived based on acute ecotoxicity data (EC, 2018; ECHA, 2008a). For Ni, the current MAQ-EQS in the EU is equal to 34 µg dissolved Ni/L (EC, 2013). However, a revised but not yet implemented MAC-EQS of 8.2 µg dissolved Ni/L has recently been proposed by the European Commission (EC, 2022).

Metal bioavailability and toxicity to aquatic organisms is influenced by the physicochemical composition of the receiving surface water (Campbell, 1995; Paquin et al., 2002). Water chemistry can affect metal toxicity via its influence on speciation or via competition interactions between certain cations and metal ions for binding at uptake sites or the sites of toxic action. For Ni, hardness (Mg^2+^ and Ca^2+^), pH and dissolved organic carbon (DOC) have been identified as the main toxicity modifying factors (e.g., Deleebeeck et al., 2007a, 2008a, 2009a). Bioavailability models, such as the biotic ligand model (BLM), integrate the effects of speciation and ionic competition to allow the prediction of metal toxicity for a given physicochemical condition (Paquin et al., 2002). For Ni, different bioavailability models are available to predict acute and chronic Ni toxicity (e.g., Deleebeeck et al., 2008a,b, 2009a; Kozlova et al., 2009; Santore et al., 2021). The current annual average–environmental quality standard and chronic PNEC for Ni are both bioavailability-based (EC, 2013) and are calculated using the chronic Ni bioavailability normalisation approach (Peters et al., 2023), which integrates the chronic Ni bioavailability models for algae, invertebrates, and fish (Deleebeeck et al., 2007a, 2008a, 2009a; De Schamphelaere, 2006). However, a similar approach to derive a bioavailability-based MAC-EQS or PNEC_intermittent_ for Ni is, to our knowledge, not yet available. However, the Scientific Committee on Health, Environmental and Emerging Risks (SCHEER), which advises the European Commission, has recently put forward that a revision of the MAC-EQS using a bioavailability normalization approach is needed (SCHEER, 2022). The development of a robust acute Ni bioavailability normalization approach to derive a MAC-EQS or PNEC_intermittent_ requires the compilation of a robust acute freshwater ecotoxicity database and the availability of validated acute bioavailability models to account for the differences in exposure chemistry across the acute ecotoxicity database.

Currently, three unique bioavailability models for predicting acute Ni toxicity to invertebrates exist: for *Daphnia magna* (Deleebeeck et al., 2008a), *Ceriodaphnia dubia* (De Schamphelaere et al., 2006), and *Daphnia pulex* (Kozlova et al., 2009). A chronic fish bioavailability model has been shown to predict acute Ni toxicity to larval and juvenile fathead minnow (*Pimephales promelas*) and juvenile rainbow trout (*Oncorhynchus mykiss*) with reasonable accuracy (Deleebeeck et al., 2007a). An algae bioavailability model, developed based on *Raphidocelis subcapitata*, is also available (Deleebeeck et al., 2009a). For these bioavailability models to be considered valid across several (nonmodel) species, the models must be sufficiently accurate in predicting acute Ni toxicity to taxonomically related species (i.e., cross-species extrapolation; EC, 2018; ECHA, 2008b). For several species, the cross-species application of the above models has already been demonstrated to be successful. For instance, the acute *D. magna* model can be used to accurately predict acute Ni toxicity to 10 different nonmodel cladoceran species (Deleebeeck et al., 2007b), whereas the algae bioavailability model was shown to accurately predict Ni toxicity to 10 European nonmodel green algae species (Deleebeeck et al., 2009b). Additionally, Peters et al. (2018) demonstrated that the algae model can be extrapolated to an Australian *Chlorella* sp, and He et al. (2023) demonstrated that the algae model can be used to accurately predict toxicity to *Raphidocelis subcapitata* in Chinese waters. On the other hand, the algae model was less accurate in predicting acute Ni toxicity to plants, and it has been reported that the chronic invertebrate models provide more accurate predictions for plants (Peters et al., 2018; Schlekat et al., 2010). The performance of the acute cladoceran models for predicting Ni toxicity to plants has not yet been evaluated.

**Figure 1.**
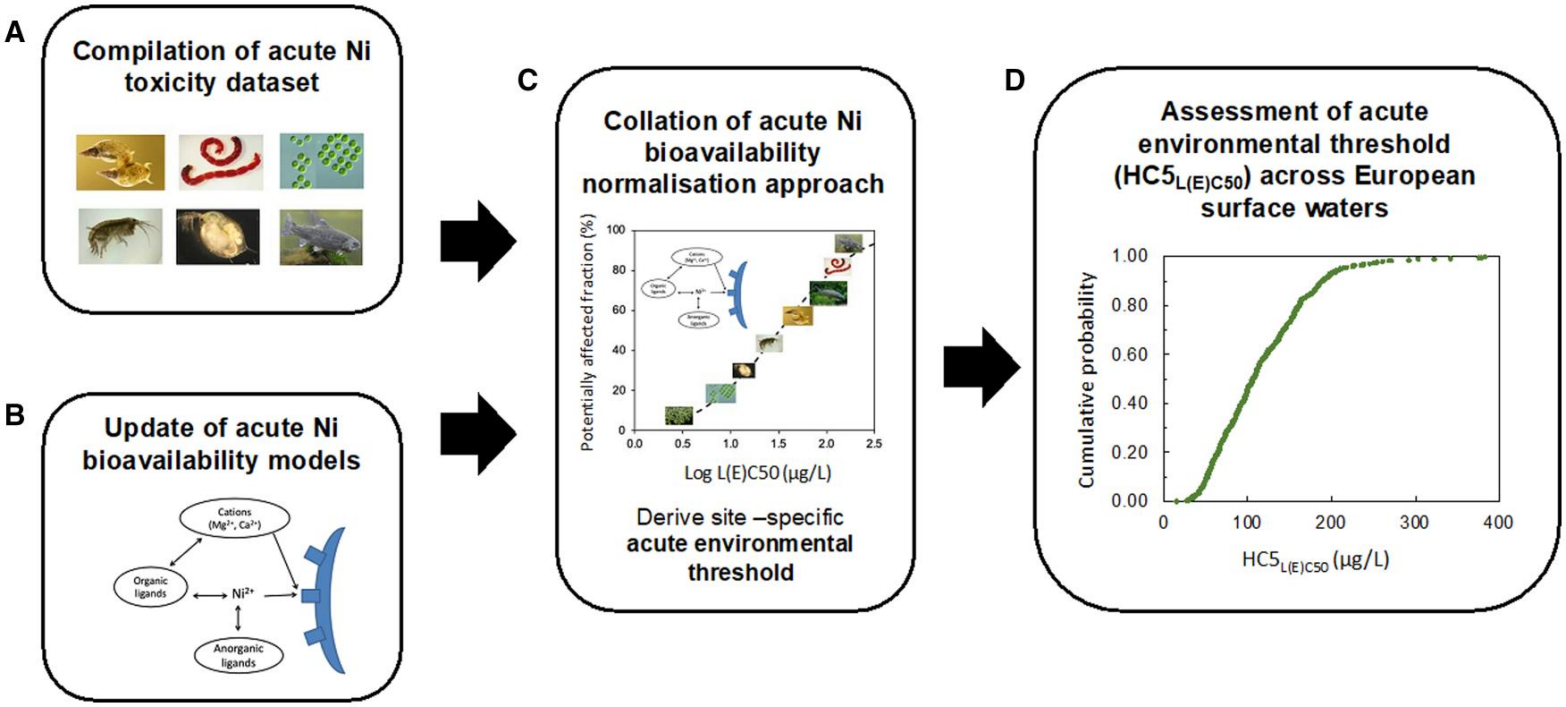
Overview of different elements considered in this study. (A) An acute Ni toxicity dataset (expressed as 50% lethal or effect concentrations (L[E]C50) for freshwater organisms was compiled from literature and study reports; (B) the acute Ni bioavailability models were updated and validated with acute Ni ecotoxicity data; (C) the acute Ni toxicity dataset was combined with bioavailability models and species sensitivity distribution (SSD) approaches into the acute Ni bioavailability normalization approach; (D) the acute Ni bioavailability normalization approach was used to assess the expected range of site-specific thresholds for acute Ni toxicity (expressed via the HC5_L(E)C50_, i.e., concentration resulting in at least 50% effect for 5% of the species in the SSD) across different European waterbodies.

This study aimed to develop an acute bioavailability normalization approach for Ni (Figure 1). For this, a high-quality acute Ni freshwater toxicity database was first collated from peer-reviewed literature. In addition, the existing acute Ni bioavailability models were reviewed and updated to extend the pH applicability ranges. Following these steps, the collated dataset was combined with the updated acute bioavailability models to generate species sensitivity distributions (SSDs) and to derive acute environmental thresholds which can serve as basis for site-specific MAC-EQS and PNEC_intermittent_. Finally, the acute Ni bioavailability normalization approach was then applied to a European reference monitoring database to characterize the expected range of site-specific acute environmental thresholds across different European waterbodies.

## Material and methods

### Compilation of an acute freshwater toxicity dataset for Ni

The acute freshwater toxicity dataset was compiled based on peer-reviewed literature and study reports (“grey literature”; queried up to 2020). Literature was screened based on reliability and relevancy criteria as described under the REACH regulation (ECHA, 2008a) and metal-specific guidance (EC, 2018; ECHA, 2008b). In short, the following selection criteria were applied. Data from approved standard test guidelines and nonstandardized tests (including standard and nonstandard species) were considered as suitable. For aquatic animals, only LC50 and EC50s (further referred to as L[E]C50) originating from acute (short-term) tests covering standard endpoints (such as mortality, immobilization, and malformation) were selected. In practice, the following exposure durations were considered short-term: 96–68 hr for vertebrates (ASTM International, 2014; Organisation for Economic Co-operation and Development [OECD], 2019), 48–168 hr for amphipods, up to 96 hr for insects, annelids, and ostracods, 48 hr for cladocerans (OECD, 2004), 96 hr for adult and juvenile molluscs (ASTM International, 2013), 48 hr for the mollusk glochidia stadia, and 24 hr for anostraca (ASTM International, 2010). For algae and plants, although the 72 hr algal and 96  and 168 hr-plant growth inhibition tests are considered chronic, the EC50 was considered as an acute value following relevant European guidance documents (EC, 2018). Data from studies with an insufficiently described methodology (e.g., test method, number of test concentrations, type of test medium used) were considered unreliable; Only measured (preferably expressed as dissolved Ni) toxicity values were selected, and nominal toxicity values were rejected. Toxicity values expressed based on total concentrations were also retained if the test was performed in artificial medium (i.e., it was assumed that the total concentration is equal to the dissolved concentration). Only Ni-only exposures with soluble nickel salts were considered relevant for the effects assessment. Studies were also rejected if indications existed that impurities or other substances might have influenced the toxic properties of the substance under investigation. Although both natural and artificial test waters representative for freshwater were accepted (ECHA, 2008a), only data for which adequate physicochemical characteristics were reported in the original study were retained. At a minimum, the parameters of pH, DOC and Ca (or hardness) needed to be reported. In addition, test medium pH and Ca need to fall within the applicability ranges of the corresponding bioavailability model (See Table 1). For bioavailability normalization, Mg, Na, K, SO_4_, Cl and dissolved inorganic carbon concentrations are also required. If these additional parameters were not reported in the original publications, these were retrieved based on nominal added values in synthetic medium or from publications using the same type of test medium. For test media where only hardness was reported, Ca and Mg concentrations were estimated based on a 3:1 ratio (on molar basis; Van Sprang et al., 2016). Inorganic carbon concentrations were estimated based on pH and alkalinity, assuming an open system (Stumm and Morgan, 1996). For synthetic test media to which no organic matter was added, DOC concentrations were set to 0 following Nys et al. (2016) and Peters et al. (2023). Background concentrations of DOC (< 1 mg/L) have been shown to not substantially influence Ni speciation calculations (De Schamphelaere et al., 2006).

**Table 1.** Overview of pH applicability range and model parameters acute Ni bioavailability models.

	Parameter	Pre-existing crustacean bioavailability models			The acute Ni bioavailability model set integrated in the acute bioavailability normalization approach to derive a site-specific HC5_L(E)C50_				
		*Daphnia magna* model^a^	*Daphnia pulex* model^b^	*Ceriodaphnia dubia* model^c^	pH extended average invertebrate model^d^		Algae model^e^		Fish model^f^
					pH^g^5.6–8.0	pH^g^8.0–8.9	pH^g^5.7–8.2	pH^g^8.2–8.7	5.5–8.8
**Model parameter**	Log K_MgBL_ (L/mol)	2.47	3.60	3.30	3.32	3.32	3.30	–	3.60
	Log K_CaBL_ (L/mol)	3.10	4.20	3.30	3.80	3.80	–	–	3.60
	S_pH_ (-)	0	0	0	0	1.01	0.143	0.906	0.324
**Bioavailability model application ranges^h^**	pH (-)	5.7–8.1	5.6–8.3	6.3–8.1	5.6–8.9		5.7–8.7		5.5–8.8
	Hardness (mg CaCO_3_/L)	6.2–292	16.0–161	15.0–253	6.2–339		6.3–315		12–290

a Deleebeeck et al. (2008a).

b Kozlova et al. (2009).

c De Schamphelaere et al. (2006).

d This study.

e Deleebeeck et al. (2009a), Nys et al. (2016).

f Deleebeeck et al. (2007a).

g For the pH extended average invertebrate model and the algae model, different bioavailability model parameters are applicable depending on the pH of the considered water.

h Physicochemical application ranges based on the calibration and validation ranges of the respective models.

*Note.* DOC = dissolved organic carbon HC5_L(E)C50_ = acute 5% hazardous concentration (i.e., the concentration that results in 50% effect for exactly 5% of the species); K_CaBL_ = biotic ligand stability constant for binding of Ca^2+^ to the Ni biotic ligand; K_MgBL_ = biotic ligand stability constant for binding of Mg^2+^ to the Ni biotic ligand; S_pH_ = slope of the log-linear relation between pH and Ni^2+^ toxicity.

### Bioavailability modeling

For all bioavailability modeling, speciation of Ni^2+^ and other ions was calculated with the software package Windermere Humic Aqueous Model VI (Tipping, 1998) following the assumptions used for the development of acute bioavailability models (e.g., Deleebeeck et al., 2007a) and in the chronic Ni bioavailability modeling approach (Peters et al., 2023; see online supplementary material Supplemental Information S1).

#### Existing acute Ni bioavailability models

The existing acute Ni bioavailability models follow two different types of model structure, that is, either a classic linear BLM-type model or a generalized bioavailability model (gBAM)-structure. In the classic linear BLM-type model, cationic competition at the biotic ligand site is incorporated as a linear effect. However, it has been observed that the effect of H^+^ on free metal ion (Me^2+^) is not necessarily linear but often exhibits a curvilinear trend (e.g., for algae and fish; Deleebeeck et al., 2007a, 2009a). As such, the effect of pH on metal toxicity is incorporated in the gBAM-structure as a log-linear relation between pH and the free Me^2+^ toxicity (ECx_Me2+_). This relationship is parametrized in the gBAM using the pH slope S_pH_. Although the original crustacean bioavailability models for *Daphnia magna* (Deleebeeck et al., 2008a), *Ceriodaphnia dubia* (De Schamphelaere et al., 2006), and *Daphnia pulex* (Kozlova et al., 2009), follow a classic linear BLM structure, the model formulations were adapted in our study from a classic linear BLM structure to the gBAM structure. Hence, all acute Ni bioavailability models (i.e., crustacean, fish, and algae models) can be mathematically expressed as follows:


(1) L(E)C50Ni2+, model y, i, predicted=10-(Q50Ni2+,model y+SpH,model y×pHi)×1+ ∑KCatzBL,model yCat2+zi

In *Equation 1*, L(E)C50_Ni2+, model y, i, predicted_ is the 50% lethal or effective concentration of Ni^2+^ expressed as free ion activity (mol/L) predicted by the corresponding model of taxon *y* in target water *i*. *Q*50_Ni2+,model_  _*y*_ is the intrinsic sensitivity under the model for taxon *y*. *S*_pH, model_  _*y*_ is the slope of the log-linear effect of pH on Ni^2+^ toxicity of the model for taxon *y* (unitless). pH_i_ is the pH of the test water or target water *i*. *K*_Cat__*Z*__BL, model_  _*y*_, is the biotic ligand binding constant (in L/mol) in the model for taxon *y* of cation *z*. (${\text{Cat}}_{z}^{2+}$)_*i*_ is the activity (mol/L) of competitive cation *z* in the test water of target water *i*.

The considered parameters may differ between the different acute bioavailability models. Competition effects of Ca^2+^ and Mg^2+^ are included in the crustacean models and the fish model, although the algae model considers only the competition effects of Mg^2+^. In the existing crustacean models, it is assumed that S_pH_ is equal to 0, as pH has not been reported to be an important toxicity modifying factor for crustaceans (e.g., Deleebeeck et al., 2008a). All model parameters of the acute Ni bioavailability models, and their applicability ranges (i.e., the physicochemical ranges over which each model has been successfully validated) are listed in Table 1.

#### Updating the bioavailability modeling procedure for invertebrates

The three existing species-specific bioavailability models for predicting acute Ni toxicity to crustaceans (i.e., *D. magna* [Deleebeeck et al., 2008a], *C. dubia* [De Schamphelaere et al., 2006] and *D. pulex* [Kozlova et al., 2009]) consider the competition effects of Ca^2+^ and Mg^2+^ at the Ni^2+^ biotic ligand site. However, the values of the biotic ligand stability constants (K_CaBL_ and K_MgBL_) differ between the different species (See Table 1). To potentially simplify the bioavailability normalization procedure for invertebrates, it was evaluated whether a newly derived unified invertebrate model (an “average invertebrate model”) could instead predict accurately acute Ni toxicity to all invertebrates. The concept of a unified invertebrate model differs from the approach that is applied to chronic Ni normalizations (see Peters et al., 2023). In the chronic approach, the prediction performance was assessed for different species or invertebrate groups (i.e., mollusk, insects, etc) in the Ni database across each of the available invertebrate models (*D. magna* vs. *C. dubia* chronic model) and the best-performing model has been carried forward as the “recommended” model for that species or taxonomic group. This approach, however, has challenges in that it is not always clear which model is best performing and, in some cases, the best-performing model differed between species within a single taxonomic group. To develop the “average invertebrate model,” the biotic ligand stability constants for Ca and Mg (K_CaBL_, K_MgBL_) of the three pre-existing crustacean models were averaged based on the arithmetic mean, giving all model parameters an equal weight. In a next step, it was also evaluated whether the assumption of the absence of a pH effect in the average invertebrate model is valid. This was done by correcting Ni^2+^ toxicity for possible differences in concentrations of the competing ions Mg^2+^ and Ca^2+^, using *Equation 2*, and plotting this value against pH.


(2) $${L(E)C50}_{Ni^{2+},\mathrm{model}\;y,i}^{**}=\frac{L(E)C50_{Ni^{2+},\mathrm{observed}\;\mathrm{species}\;k,\;i}}{1+\sum K_{\mathrm{Ca}t_{z}BL,\mathrm{model}\;y}{\left\{{{\mathrm{Cat}}^{2+}}_{z}\right\}}_{i}}$$

In *Equation 2*, ${L(E)C50}_{Ni^{2+}}^{**}$ is the Ni^2+^ toxicity, expressed as free Ni^2+^ activity (mol/L) for species k in test medium *i* which has been corrected for competition effects of Mg^2+^ and/or Ca^2+^ using the bioavailability model for taxon *y*.

#### Validation of the average invertebrate model

The prediction performance of the final average invertebrate model was evaluated using a dataset covering studies that evaluated the effect of toxicity modifying factors on acute Ni toxicity for 15 invertebrate species, including not only *D. magna*, *D. pulex* and *C. dubia* but also 10 other cladocerans, an annelid, and an amphipod. For the validation, relevant bioavailability studies published by 2020 were considered. Studies were considered relevant for inclusion in the cross-species validation if acute Ni toxicity in at least two media with different physicochemical conditions were evaluated. Datasets considering only the effects of natural organic matter while keeping all other physicochemical parameters constant were disregarded, as these do not allow the validation of the entire bioavailability model. A detailed overview of the different datasets used is given in online supplementary material Supplemental Information S2.1. To validate the average invertebrate model, the intrinsic sensitivity parameter (Q50) for a given species was calibrated for each dataset separately (using the arithmetic mean Q50 of a dataset) to account for possible shifts in Ni sensitivity due to inter- and intralaboratory differences (e.g., clone difference, shifts in sensitivity over time) using the equations included in online supplementary material Table S2.2.

#### Cross-species validation of the acute bioavailability models for higher plants

Because there remains uncertainty on which model is best suited to predict bioavailability effects to higher plants, it was evaluated whether the average invertebrate acute model or the pre-existing algae model (using the pH extended algae model of Nys et al., 2016) predicts Ni toxicity to plants most accurately. For this evaluation, four datasets covering two species (*Lemna minor* and *Lemna aequinoctalis*) were used (See online supplementary material Supplemental Information S2). As in the validation approach for the invertebrates, the intrinsic sensitivity (Q50) of both models was again recalibrated for each species and dataset separately using the equations listed in online supplementary material Table S2.2 (average invertebrate model) and Table S2.3 (algae model).

#### Model performance evaluation

The evaluation of the prediction performance considered the recommendations of Garman et al. (2020) and followed the approach previously used by Besser et al. (2021) with one adaptation. This approach considers three different metrics in the evaluation of the prediction performance of a bioavailability model, which are combined in an overall model performance score (MPS). The MPS considers *r*^2^, the coefficient of determination, which represents the goodness-of-fit of the bioavailability model predicted toxicity relative to the observed toxicity; the factor of agreement (FA), which represents the fraction of toxicity data predicted within a 2-fold error; and the total residual score (Tot RS), which is a statistic representing the bias in the model predictions relative to the main toxicity modifying factors considered in the model. The *r*^2^-statistic is calculated from the ratio of the sum of squares of the residuals (SSR; comparing log_10_-transformed predicted L[E]C50 vs. log_10_-transformed observed L[E]C50) to the total sum of squares (SST; defining the overall residual variation in log_10_-transformed observed L[E]C50), using *Equation 2* in Besser et al. (2021). The *r*^2^ value can be negative when the residual score of the model predictions is higher compared with that of the observed values (i.e., SSR>SST). In that case, for the purpose of this study, the *r*^2^ was set to 0. An *r*^2^ of 0 indicates that the bioavailability model is not performing better than the null-model that uses the mean L(E)C50 from the considered dataset as a predictor. This adaptation from the approach of Besser et al. (2021) was made because a negative score can possibly result in a negative overall MPS, whereas the adaptation results in that all three metrics included in the MPS vary between 0 and 1. The Tot RS was calculated using *Equation 3* in Besser et al. (2021) and represents the overall bias in the relationship between model residuals and specified water chemistry variables. For this study, only toxicity modifying factors of pH, DOC, Ca and/or Mg were considered. The final MPS-score was calculated as the average between *r*^2^, FA, and Tot RS. The MPS score is a value between 0 and 1, with the higher the MPS indicating a better performance of the evaluated bioavailability model. An MPS-score was calculated for each dataset separately.

The MPS score can also be used to compare model performance between different bioavailability models for one dataset and as such was used to compare the species-specific bioavailability models versus the average invertebrate model for the crustacean model-species (i.e., *C. dubia*, *D. magna*, and *D. pulex*) or between the pH extended-algae model and the pH extended-average invertebrate model for the plant species.

### Development of the acute Ni bioavailability normalization approach

In the acute Ni bioavailability normalization approach, the acute Ni toxicity dataset is combined with the acute bioavailability model set and applied to an SSD approach. The selected acute Ni toxicity dataset and the selected acute bioavailability models will be discussed in more detail in the *Results and discussion* section.

The SSD approach includes both species-averaging and the fitting of a distribution to the normalized species-averaged toxicity data. Species-averaging is performed to avoid overrepresentation of ecotoxicity data from one or several species (ECHA, 2008a,b). If the toxicity dataset contains multiple entries for a species, toxicity was averaged (arithmetic mean) for that species at the intrinsic sensitivity level (Q50) following Peters et al. (2023). If, for one species, multiple endpoints are available in the dataset, the (averaged) intrinsic sensitivity resulting from the most sensitive endpoint was selected (i.e., the highest Q50 value). If multiple exposure durations were available for one species (e.g., *Hyalella azteca*), the lowest LC50 has been selected. The species-averaging approach was not differentiated between different life-stages of a particular species, that is, intrinsic sensitivities were averaged over all life-stages of a species. This choice was made because there was not always detailed information available on the life stage of the organisms for all data entries.

In the final step of normalization, a distribution is fitted to the normalized dissolved concentrations (one L[E]C50 value per species in the SSD) to calculate an HC5 specific to the target water. In the context of the intermittent releases environmental threshold derivation (MAC-EQS and PNEC_intermittent_), the HC5 refers to the concentration resulting in at least 50% effect for 5% of species in the SSD (abbreviated as HC5_L(E)C50_, also referred to as the acute environmental threshold). For each target water, a separate normalized SSD is constructed by fitting six different distributions (gamma, log-normal, logistic, normal, Weibull, and Gumbel) to the log_10_-transformed normalized toxicity data in R (Ver. 1.3.1093) using the “fitdistrplus”-package (Delignette-Muller et al., 2022). The selected set of distributions is based on those recommended in SSD-approaches across several jurisdictions around the globe (Fox et al., 2021). The Anderson-Darling statistic has been used to select the best-fitting distribution for each target water (ECHA, 2008a) as it gives more weight to the tails of the distribution, which are the regions of interest for PNEC or EQS derivation. The median HC5_L(E)C50_ are derived from the best-fitting distribution for each site-specific HC5_L(E)C50_. The sampling uncertainty was considered using the 90% confidence interval (represented by HC5-5 and HC5-95) for the best-fit distribution using parametric bootstrap simulation of the normalized geomean L(E)C50 with replacement (Verdonck et al., 2001).

The acute Ni bioavailability normalization approach was applied to two reference datasets in European freshwater bodies to give an overview of the distribution of site-specific HC5_L(E)C50_ values for European waterbodies. The first one, the “ecoregion”set, represents a regulatory set of seven specific freshwater scenarios that may occur in Europe and for which physicochemical conditions are within the general boundaries of metal bioavailability models (ECHA, 2008b). The ecoregion set has been the basis of the derivation of the chronic PNEC for Ni (EC, 2008b). Detailed physicochemistry of the ecoregion set used for bioavailability modeling is available in online supplementary material Supplemental Information S3. A second reference dataset, the Forum of the European Geological Surveys (FOREGS) dataset (Salminen et al., 2005) was selected to reflect the possible physicochemical conditions more broadly in European water bodies. Only the surface waters for which physicochemistry is within the application range of the acute Ni bioavailability normalization approach (See the *Results and discussion* section) were used for acute environmental threshold derivation.

## Results and discussion

### Acute Ni toxicity dataset

The acute Ni toxicity dataset was compiled based on a literature screening for high-quality acute Ni toxicity data for freshwater organisms. In total, 449 unique toxicity data entries were identified covering 63 different freshwater species. These species include 13 algae, four amphibians, two amphipods, one annelid, 14 cladocerans, three anostraca, eight fish, two higher plants, three insects, 12 mollusks, and one ostracod. Toxicity data covers the following endpoints: growth rate, yield, and cell density for algae; mortality and malformation for fish and amphibians; mortality and/or immobilization for invertebrates, and growth rate (based on frond number) and frond count for plants. All retained data are based on measured Ni concentrations (either total or dissolved), with most data (389 out of 449 data entries) based on dissolved concentrations. Within the toxicity database, pH ranges between 5.5 and 8.9 (median 7.5), hardness between 6.2 and 339 mg CaCO_3_/L (median 77 mg CaCO_3_/L), and DOC between 0 and 39 mg DOC/L (median 0 mg/L, as it was assumed that DOC concentrations in synthetic media were equal to 0; see *Compilation of an acute freshwater toxicity dataset for Ni* section). Nonnormalized effect concentrations ranged by five orders of magnitude, with L(E)C50 ranging between 7.8 µg dissolved Ni/L (96-hr growth rate for the duckweed *Lemna aequinoctalis*) and 477,000 µg dissolved Ni/L (48-hr mortality for the amphipod *Gammarus pulex*). The 5th to 95th percentile of nonnormalized L(E)C50 ranged from 67 µg dissolved Ni/L to 14 656 µg dissolved Ni/L. The entire database with retained ecotoxicity data, including detailed information of physicochemical parameters used for bioavailability modeling, appears in the online supplementary material as part of the acute Ni bioavailability normalization tool (See online supplementary material Supplemental Information S4). The tool also contains a list of nonretained data entries, including an explanation on the reason of rejecting.

The acute Ni toxicity dataset covers the minimum data requirements for species sensitivity distribution extrapolation set out in the WFD (EC, 2018) and under the REACH guidance (ECHA, 2008a). The dataset covers both standard and nonstandard test species and includes all species that have been identified as sensitive species in the recent chronic Ni database update (e.g., *Ceriodaphnia dubia*, *Lymnaea stagnalis*, *Hyalella azteca*, and *Lemna* species; Peters et al., 2023).

### Bioavailability modeling

#### Updating the acute Ni invertebrate bioavailability modeling approach

The three existing acute Ni crustacean models for *D. magna* (Deleebeeck et al., 2008a), *D. pulex* (Kozlova et al., 2009), and *C. dubia* (De Schamphelaere et al., 2006) differ in the magnitude of the competition effect of Ca^2+^ and Mg^2+^ (i.e., the biotic ligand stability constants; Table 1). The average invertebrate model was developed by averaging the biotic ligand stability constants of the three pre-existing crustacean models with resulting log_10_K_MgBL_ and log_10_K_CaBL_ equal to 3.32 and 3.80 (log L/mol), respectively.

**Figure 2.**
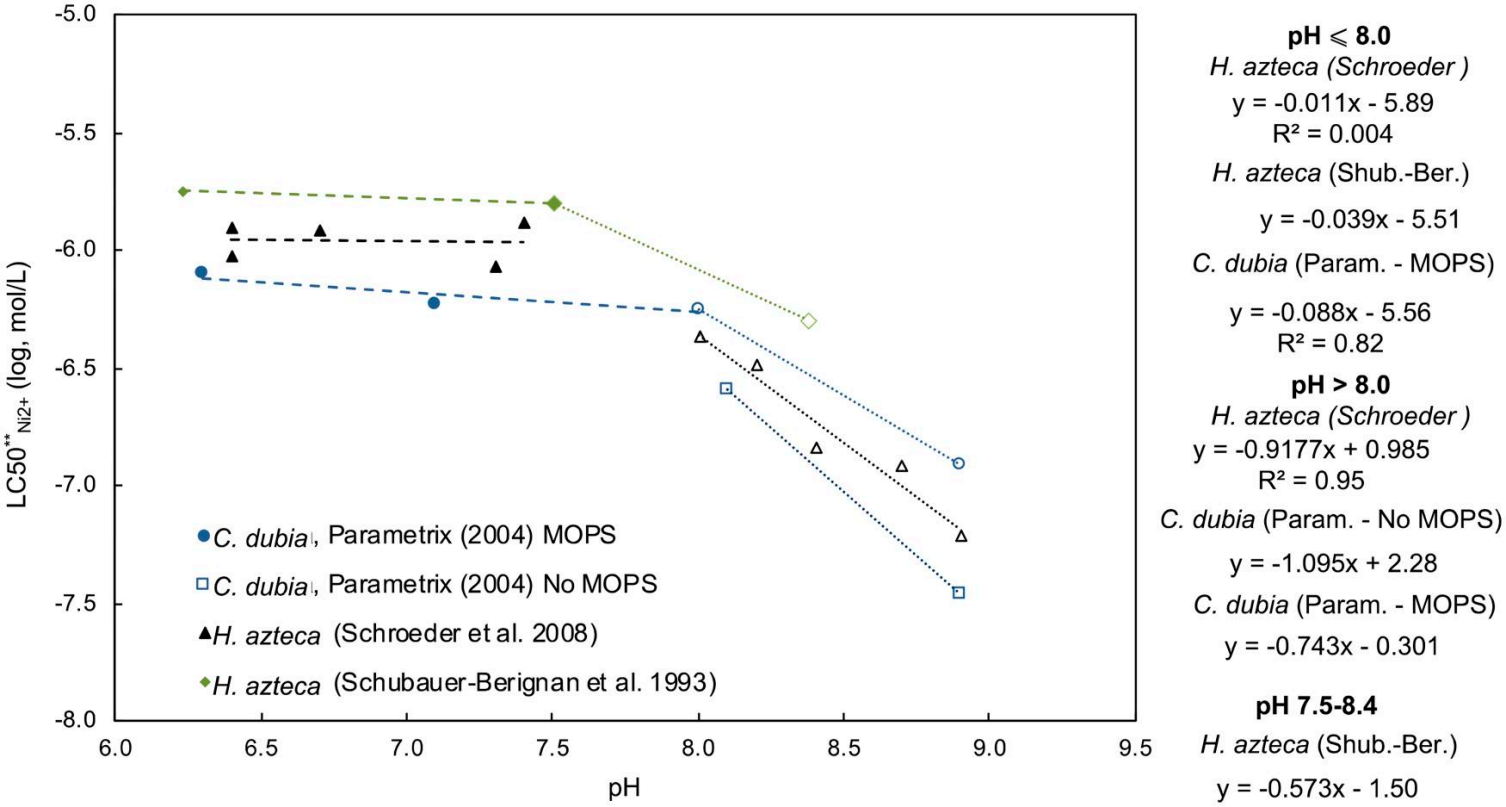
Observed log_10_ 50% lethal concentrations corrected for competition effects of Mg^2+^ and Ca^2+^ (LC$50_{\text{Ni}2+}^{**}$; *Equation 3*) as a function of pH for *Ceriodaphnia dubia* (circles and squares) and *Hyalella azteca* (triangles and diamonds). Filled symbols indicate toxicities observed in test media with pH up to pH 8.0, open symbols represent toxicities observed in test media with pH greater than and including 8.0. Linear relationship between pH and logLC50**_Ni2+_ are plotted with a dashed line for the relationship less than pH 8.0 and a dotted line for pH greater than 8.0. Data are taken from Parametrix (2004; 48 h; *C. dubia*), Schroeder et al. (2010; 7d; *H. azteca*), and Schubauer-Berigan et al. (1993; 96 h; *H. azteca*). Equations of the linear relationship between pH and log_10_LC$50_{\text{Ni}2+}^{**}$ are given at the right of the graph. Correction for competition effects was done using the parameters of the average invertebrate model. MOPS = 3-(*N*-morpholino)propanesulfonic acid.

For two studies, the effect of pH on Ni^2+^ toxicity at high pH (pH > 8.0) was investigated more closely: the *C. dubia* dataset of Parametrix (2004) and the *H. azteca* dataset of Schroeder et al. (2010). Figure 2 shows the effect of pH on Ni^2+^ sensitivity corrected for Ca^2+^ and Mg^2+^ (L(E)C50**_Ni2+_; *Equation 2*) for these datasets. For both *C. dubia* (circles and squares) and *H. azteca* (triangles), only a marginal effect of pH on the competition-corrected Ni^2+^ sensitivity is observed less than a pH of approximately 8.0; greater than pH 8.0, the competition-corrected Ni^2+^ sensitivity decreases clearly with increasing pH. A third dataset, reporting on univariate effects of pH on acute Ni toxicity to *H. azteca* (Schubauer-Berigan et al., 1993), corroborates this general observation of the absence of a pH effect at low pH (pH range 6.2–7.5), whereas there is a clear increase in Ni^2+^ toxicity in the pH range between 7.5 and 8.4 (green diamonds in Figure 2). In addition, Deleebeeck et al. (2008a) reported that acute Ni^2+^ toxicity to *D. magna* was not affected in the pH range between 5.7 to 7.5, but a further increase in pH to pH 8.1 resulted in an increase in Ni^2+^ toxicity. The slope of the log-linear relationship between pH and the corrected Ni^2+^ sensitivity (i.e., S_pH_) greater than pH 8.0 is 1.095 for *C. dubia* (based on the test series without 3-(N-morpholino)propanesulfonic acid [MOPS] addition) and 0.918 for *H. azteca* (Figure 2). These pH slopes are similar to the pH slopes (i.e., S_pH_) for chronic Ni toxicity to *D. magna*, *B. calyciflorus*, *C. dubia*, and *L. stagnalis* reported by Nys et al. (2016) and De Schamphelaere et al. (2006), which range between 0.996 and 1.887 in the pH range between 8.2 and 8.7.

Nys et al. (2016) suggested that the slope of the log-linear relationship between pH and chronic Ni^2+^ toxicity (i.e., S_pH_) differs depending on the considered pH range, with pH slopes more than approximately pH 8.2 being much steeper than less than pH 8.2 (De Schamphelaere et al., 2006; Nys et al., 2016). The latter is in line with the observations for acute Ni toxicity in this study. Several explanations for the increased effect of pH on Ni^2+^ toxicity at high pH have previously been put forward, including the presence of multiple types of biotic ligand sites that may be singly or doubly protonated (Nys et al., 2016), the bioavailability of complexes such as Ni(OH)_2_ and Ni(CO_3_)_2_^2−^ that become increasingly more important compared with the free Ni^2+^ ion at high pH (Deleebeeck et al., 2008b), or alternatively, dissociation of Ni^2+^ from these complexes in the microenvironment surrounding the biotic ligand (Deleebeeck et al., 2008b). Another possible explanation is that greater than pH 8.0, a change in the cell membrane permeability can occur, increasing the uptake of Ni(-complexes; Lavoie et al., 2012). Recently, Santore et al. (2021) and Besser et al. (2021) have suggested that at high pH, Ni toxicity may be affected by bicarbonate toxicity or by the mixture effects between Ni and bicarbonates that may occur under these conditions.

The absence of a pH effect below pH 8.0 on acute Ni^2+^ suggests that at pH < 8.0, pH is not an important toxicity modifying factor. As such, it was previously also not considered in the existing acute crustacean bioavailability models. This differs from the chronic toxicity models for *C. dubia* and *D. magna*, (Deleebeeck et al., 2008b; De Schamphelaere et al., 2006; respectively) where pH has been incorporated as a significant toxicity modifying factor across the entire pH applicability range (although also dependent on the considered pH-level; see next section). Similar observations of a more important pH effect in chronic exposures compared with acute exposures have been reported for Cu toxicity to *D. magna* (De Schamphelaere & Janssen, 2004). It has been suggested that this difference between acute and chronic exposures is because acclimation processes become more important during prolonged exposure and, as such, may influence the relationship between toxicity modifying factors and toxicity (De Schamphelaere & Janssen, 2004).

In the chronic Ni bioavailability models, the bimodal pattern in pH slope has been integrated as a two-step procedure (Nys et al., 2016). In a first step, chronic Ni toxicity is normalized to pH 8.2 with the standard bioavailability models. In a second step, this chronic Ni toxicity is then further normalized to pH > 8.2 using the high pH slope. A slightly simpler approach can be followed to include the pH effect on Ni^2+^ toxicity in the pH extended-acute average invertebrate model, as there is no substantial pH effect up to pH 8.0. In the pH extended-average invertebrate bioavailability model, the S_pH_ is dependent on the pH range (Table 1). At pH up to and including 8.0, the S_pH_ is set to 0 (S_pH_ = 0), whereas the high pH extension of the average invertebrate model is applied in the range between 8.0 and 8.9. The proposed S_pH_ parameter to be taken forward in the high pH extended-average invertebrate model is the average of the S_pH_ of *C. dubia* (based on the dataset without MOPS; S_pH, C. dubia_ = 1.095) and *H. azteca* (S_pH, H.azteca_ = 0.9177), i.e., S_pH, average invertebrate_ = 1.006. The average invertebrate model follows the general model structure for the Ni bioavailability models (*Equation 1*), with slight modifications to implement the high pH slope. Model equations for the proposed average invertebrate model are shown in online supplementary material Table S2.2. The precise pH level where pH starts to affect acute Ni^2+^ toxicity is difficult to determine, because only a limited number of pH levels have been considered in each test series. However, the combined evidence based on the *C. dubia* dataset of Parametrix (2004) and the *H. azteca* dataset of Schroeder et al. (2010) suggest this occurs at approximately pH 8.0 (Figure 2). Hence, it is suggested that the pH effect is implemented greater than pH 8.0 and up to a pH of 8.9. It should be noted that this approach assumes that competition of Ca^2+^ and Mg^2+^ at the Ni^2+^ biotic ligand is independent of the pH level. This assumption cannot be evaluated based on the available data.

#### Validation of the pH extended-average invertebrate model

**Figure 3.**
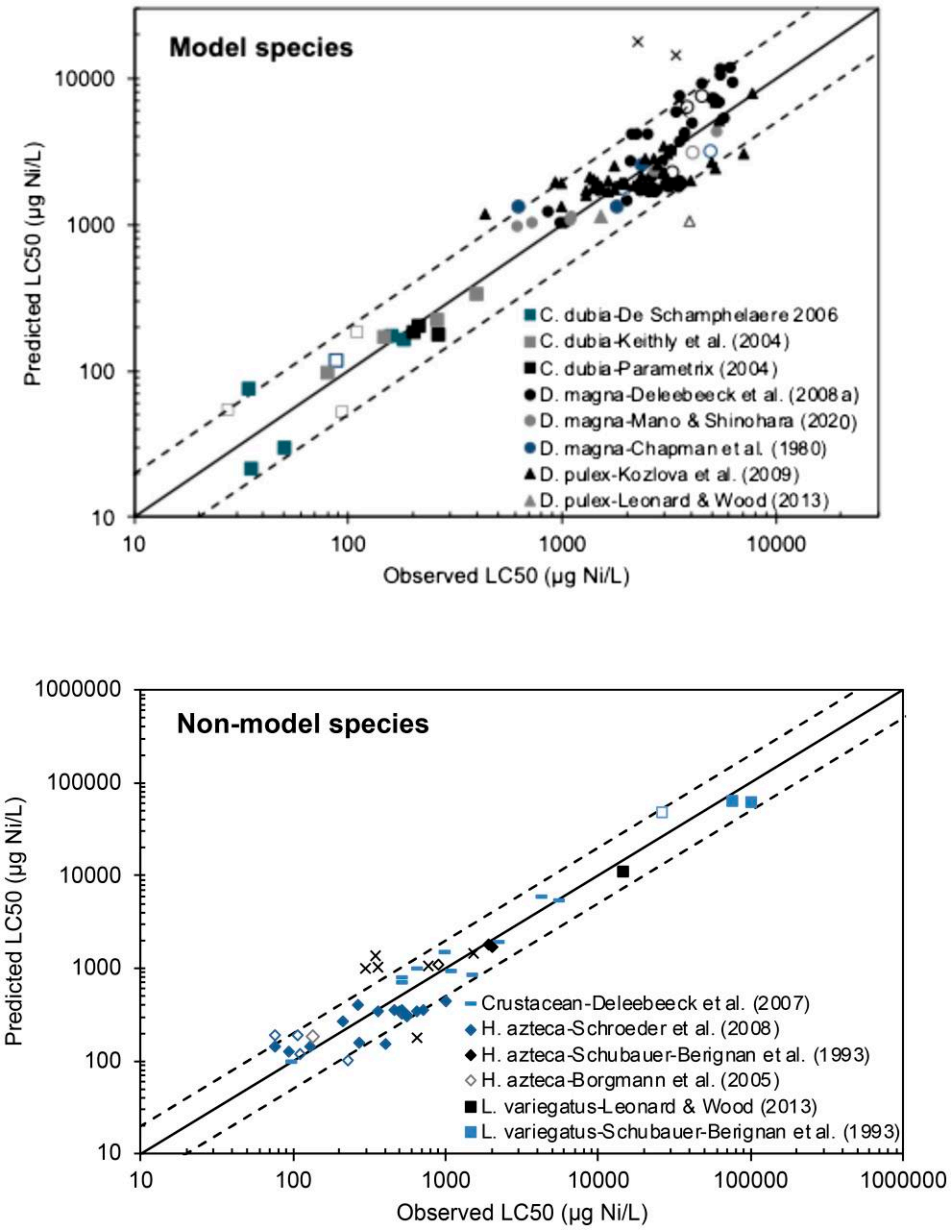
Observed versus predicted 50% lethal concentrations (LC50; µg dissolved Ni/L) to both model species (upper panel) and nonmodel species (lower panel) predicted with the “pH extended-average invertebrate model.” The full line represents the perfect prediction line, dashed lines indicate a twofold prediction error on the observed Ni toxicity; open symbols represent toxicity data obtained in test solutions with pH > 8.0; X symbols indicate that toxicity data has been obtained in test solution with high hardness (> 290 mg CaCO_3_/L). Intrinsic sensitivities have been calibrated for each dataset separately, based on all waters with Ca and Mg < 3 mM.

Details on model equations (See online supplementary material Table S2.2) and the dataset-specific intrinsic sensitivities of the average invertebrate model are given in the online supplementary material (See online supplementary material Tables S2.4–S2.5). Figure 3 shows the predictive performance of the pH extended-average invertebrate model, and MPS are summarized in Table 2. The average invertebrate model, when calibrated for each species and dataset separately and applied within the combined calibration ranges of the original crustacean models, predicted acute Ni toxicity to model species for most datasets with reasonable accuracy (i.e., FA ≥ 0.80), which shows that the pH extended-average invertebrate model can predict acute Ni toxicity to invertebrates within a twofold error for most datapoints. When evaluating the overall model performance score, most datasets (10 out of 14) resulted in an overall MPS ≥ 0.70. Although there are currently no clearly defined thresholds for the evaluation of model performance based on MPS, the MPS gives an indication of overall prediction performance, goodness-of-fit, and bias in model predictions. Low MPS scores were observed for two *D. magna* datasets with MPS of 0.50 and 0.60 (datasets of Chapman et al. [1980] and Deleebeeck et al. [2008a], respectively), an *L. variegatus* dataset with MPS of 0.69 (datasets of Schubauer-Berigan et al. [1993]) and the crustacean dataset of Deleebeeck et al. (2007b; average MPS 0.64). Low MPS scores were mainly related to the *r*^2^ statistic, which was the most sensitive metric within the MPS-calculation. The *r*^2^ represents the goodness-of-fit of the bioavailability model relative to a null model, assuming that variation is random. Across datasets, *r*^2^ varied between 0.00 and 0.95. Very low *r*^2^-scores were observed for the *D. magna*-dataset of Deleebeeck et al. (2008a; *r*^2^ = 0.00) and the *D. magna* dataset of Chapman et al. (1980; *r*^2^ = 0.04). A possible explanation for the low *r*^2^ scores in some of these datasets is the high intertreatment variability, for example, Chapman et al. (1980) reported a threefold difference in LC50 in repeated tests in well water at a hardness of 50 mg CaCO_3_/L (See online supplementary material Supplemental Information S4; acute toxicity dataset) with LC50s ranging between 1801 µg/L and 628 µg/L. This random variability is not captured by a bioavailability model, which typically assumes that random variability in acute LC50s of repeated tests is within a factor of two (Meyer et al., 2018; Price et al., 2022). For the *D. magna* dataset of Deleebeeck et al. (2008a), the low *r*^2^-score can be explained by the overestimation of the magnitude of Ca^2+^ and Mg^2+^-effects on acute Ni^2+^ toxicity in the average invertebrate model compared with the actual observed effects in this dataset (See online supplementary material Figure S2.1). This specific dataset was used to parametrize the acute *D. magna* bioavailability model (Deleebeeck et al., 2008a), with log_10_K_CaBL_ and log_10_K_MgBL_ 1.2-fold and 1.3-fold lower than those of the pH extended-average invertebrate model (Table 1). Based on the residual scores for the different toxicity modifying factors, the prediction bias is mainly related to Ca, with RS Ca equal to 0.61 for the average invertebrate model (See online supplementary material Table S2.7). Similarly, the lower overall MPS-score for the crustacean dataset of Deleebeeck et al. (2007b; average 0.64, range: 0.23–0.99) can be attributed to differences in the magnitude of the hardness effect on acute Ni toxicity across crustaceans (See online supplementary material Figure S2.2).

**Table 2.** Performance of the species-specific model^a^ and the average invertebrate model for different invertebrate species.

Species	Reference^a^	Test duration	Toxicity modifying factor^b^ (No. of samples)	Species-specific model^c^				Average invertebrate model			
				*r* ^2^ ^d^	FA^e^	Tot RS^f^	MPS^g^	*r* ^2^ ^d^	FA^e^	Tot RS^f^	MPS^g^
***Ceriodaphnia dubia***	[1]	48 h	Natural water (*N* = 6)	0.84	0.83	0.91	0.86	0.83	0.83	0.77	0.81
	[2]	48 h	Hardness (*N* = 4)	0.81	1.00	0.66	0.82	0.90	1.00	0.78	0.89
	[3]	48 h	pH (*n* = 4 [pH ≤ 8.1]; *N* = 6 [pH ≤ 8.9])	0.00	0.75	0.82	0.52	0.59	1.00	0.96	0.85
***Daphnia magna***	[4]	48 h	Ca, Mg, Na and pH, Natural water (*N* = 52)	0.55	0.98	0.92	0.82	0.00	0.94	0.85	0.60
	[5]	48 h	Hardness, pH (*N* = 5)	0.21	1.00	0.87	0.69	0.04	0.80	0.67	0.50
	[6]	48 h	Natural water + pH (*N* = 10)	0.63	0.90	0.78	0.77	0.89	1.00	0.85	0.91
***Daphnia pulex***	[7]	48 h	Ca, Mg, K, Na, pH, DOC (*N* = 44)	0.59	0.91	0.97	0.82	0.50	0.86	0.94	0.76
	[8]	48 h	Hardness (*N* = 2)	0.97	1.00	0.78	0.91	0.89	1.00	0.61	0.83
***Hyalella azteca***	[9]	7 d	Hardness (*N* = 2)	NA	NA	NA	NA	0.33	1.00	0.85	0.72
	[10]	7 d	Hardness, pH, alkalinity (*N* = 17; [pH ≤ 8.3]; *N* = 21 [pH ≤ 8.9])	NA	NA	NA	NA	0.59	0.90	0.86	0.79
	[11]	96 h	pH (*n* = 2; [pH ≤ 8.3]; *N* = 3 [pH ≤ 8.9])	NA	NA	NA	NA	0.82	1.00	0.94	0.92
***Lumbriculus variegatus***	[8]	96 h	Hardness (*N*= 2)	NA	NA	NA	NA	0.95	1.00	0.94	0.96
	[11]	96 h	pH (*N* = 3)	NA	NA	NA	NA	0.37	1.00	0.71	0.69
**10 Crustacean species**	[12]	48 h	Hardness (*N* = 2)	NA	NA	NA	NA	0.45^h^ (0.00–1.00)	0.95^h^ (0.50–1.00)	0.61^h^ (0.19–0.97)	0.64^h^ (0.23–0.99)

a References: [1] De Schamphelaere et al. (2006); [2] Keithly et al. (2004); [3] Parametrix (2004); [4] Deleebeeck et al. (2008a); [5] Chapman et al. (1980); [6] Mano and Shinohara (2020); [7] Kozlova et al. (2009); [8] Leonard and Wood (2013); [9] Borgmann et al. (2005); [10] Schroeder et al. (2010); [11] Schubauer-Berigan et al. (1993); [12] Deleebeeck et al. (2007b).

b Potential toxicity modifying factors (TMF) considered in the dataset. “Natural water” indicates that a set of natural waters with different physicochemical conditions has been evaluated. Note that only waters within the applicability ranges of the considered bioavailability model have been considered for calculating performance scores.

c For model-species the species-specific model indicates that the *C. dubia* model was used for all *C. dubia*-datasets, the *D. magna* model was used for all *D. magna*-datasets and the *D. pulex* model was used for all *D. pulex*-datasets.

d *r* ^2^ is a metric for the goodness-of-fit of the bioavailability model relative to a null-model in which it is assumed that all variability among toxicity data is attributable to random variation and not to differences in TMF.

e Factor agreement (FA) represents the fraction of toxicity data predicted within twofold error.

f Total residual score (Tot RS) represents bias in the model predictions relative to the main toxicity modifying factors of the bioavailability model (pH, DOC, Ca, and Mg) considered in the dataset.

g Model performance score (MPS) represents the average of *r*^2^, FA and Tot RS. MPS is a value between 0 and 1, the higher the MPS the better the performance of the evaluated bioavailability model.

h Average score across 10 different crustacean species tested in 24 test media reported, minimum and maximum scores reported in brackets.

*Note.* h = hours, d = days, DOC = dissolved organic carbon, NA = not applicable.

The total residual scores (Tot RS) varied between 0.61 and 0.96. Whereas Tot RS-scores were generally greater than 0.70, lower Tot RS (< 0.70) were observed for three datasets (see Table 2). Low Tot RS-scores indicate there may be (considerable) bias in model predictions relative to one or more toxicity modifying factors in the average invertebrate model. Low RS scores can be mostly related to the variance of the hardness effect between different invertebrate species, (e.g., for the crustacean dataset of Deleebeeck et al., 2007b; average Tot RS 0.64; see online supplementary material Figure S2.2) and between datasets of a specific species (e.g., the *D. pulex*-dataset of Leonard & Wood (2013); Tot RS = 0.61; see online supplementary material Figure S2.3).

For the crustacean species for which a species-specific bioavailability model is available (i.e., *D. magna*, *D. pulex*, and *C. dubia*), the predictive performance of the pH extended-average invertebrate model was demonstrated to be similar (MPS within 10%) or better for all *C. dubia* and *D. pulex*-datasets (Table 2; see online supplementary material Figure S2.4) when compared with the species-specific model. For *D. magna*, the predictive performance of the pH extended-average invertebrate model was higher for the dataset of Mano and Shinohara (2020; Table 2; see online supplementary material Figure S2.4), but lower for the dataset of Deleebeeck et al. (2008a) and of Chapman et al. (1980).

For the two datasets that include media tested at more extreme pH (pH up to 8.9: i.e., *C. dubia* data of Parametrix [2004] and the *H. azteca* data of Borgmann et al. [2005]), the overall model prediction performance (MPS) of the pH extended-average invertebrate model increased compared with that of the average invertebrate model that would not include a pH extension (See online supplementary material Table S2.8 and Figure S2.5). For those datasets including test solution with pH greater than 8.0 but less than 8.4, the pH extended-average invertebrate model results in prediction performances similar to or higher than an average invertebrate model without pH extensions. The only exception to this was the dataset of Chapman et al. (1980) for which MPS decreased from 0.78 to 0.50 (average invertebrate model vs. pH extended-average invertebrate model). For the datasets with pH less than 8.0, the pH extended-average invertebrate model results in the same model prediction performance as the average invertebrate model.

Given that the fraction of datapoints predicted within twofold error is relatively high across datasets (FA ≥ 0.8) and the residual bias relative to the toxicity modifying factors is for most datasets relatively low (Tot RS > 0.7), it is recommended to use the pH-extended-average invertebrate model as the default model for normalization of acute invertebrate toxicity data.

#### Cross-species evaluation of the acute Ni bioavailability models for aquatic plants

It has previously been suggested that, for aquatic plants, the protective effect of Ca^2+^ on Ni^2+^ toxicity is likely stronger than for algae (Schlekat et al., 2010). For algae, no obvious Ca^2+^ effect has been observed on Ni^2+^ toxicity to *P. subcapitata* (Deleebeeck et al., 2009a). As such, there is no Ca^2+^ effect integrated in the algae bioavailability model. Given that for plants, Ca^2+^ may compete with Ni^2+^ for uptake at the Ni biotic ligand site (Schlekat et al., 2010), the chronic invertebrate models, which all integrate both a Ca and Mg biotic ligand constant, have been shown to predict toxicity to *L. minor* more accurately than the algae bioavailability model (Peters et al., 2023; Schlekat et al., 2010). In the chronic bioavailability model approach, the toxicity data for plants are therefore normalized with an invertebrate bioavailability model rather than the algae model (Nys et al., 2016; Peters et al., 2023). The protective effects of Ca on Ni toxicity have mainly been related to its role as regulator of membrane stability and ion transport through the paracellular pathway in multicellular organisms such as daphnids (Deleebeeck et al., 2008b). The absence of tight junctions in unicellular algae may be the explanation why Ca has only minor effects on Ni^2+^ uptake and toxicity to algae compared with Mg (Deleebeeck et al., 2009a; Worms & Wilkinson, 2007), and why it is not a major toxicity modifying factor for algae as opposed to multicellular plants.

In this study, we evaluated whether a similar approach of using the acute invertebrate model rather than the algae model for predicting acute Ni toxicity to higher plants is valid. Overall, considerable variability in prediction performance of the pH extended-average invertebrate model and pH extended-algae model between species (*Lemna minor* vs. *Lemna aequinoctalis*) was observed. In addition, this variation was also observed between datasets for the same species (based on *L. minor*; see online supplementary material Figure S2.6 and Table S2.10). For only one of the four datasets for plant species, the pH extended-average invertebrate model performed markedly better than the pH extended-algae model, that is, for the *L. minor* dataset of Schlekat et al., 2010 (MPS 0.93 vs. 0.51), with high scores across the different MPS statistics: *r*^2^ (0.92), FA (1.00), and Tot RS (0.87). None of the bioavailability models resulted in MPS scores greater than 0.60 for the other three datasets reporting on Ni toxicity to plant species, and all resulted in low scores for the goodness-of-fit statistic (*r*^2^) across datasets (*r*^2^ < 0.5; see online supplementary material, Supplemental Information for more detailed discussion). When considering the fraction of toxicity data predicted within twofold error, the *L. minor* dataset of Gopalapillai et al. (2013) and Nys et al. (2016) were predicted with reasonable accuracy using both bioavailability models (i.e., at least 85% of the toxicity datapoints were predicted within twofold error), whereas the *L. aequinoctalis* dataset (Peters et al., 2018) scored also low on this parameter (FA = 0.50). Finally, the prediction bias relative to the toxicity modifying factors (Tot RS) was dependent on the considered dataset and bioavailability model, with relatively low scores for the pH extended-average invertebrate model across all datasets (Tot RS ≤0.60), except for the dataset of Schlekat et al. (2010). Bias in predictions was related to either Ca, Mg and/or pH, depending on the considered dataset and bioavailability model (See online supplementary material Table S2.11).

It has been reported that bioavailability effects within the *L. aequinoctalis* dataset are difficult to predict with the chronic Ni bioavailability models (Peters et al., 2018). This may suggest that this species does not follow the general bioavailability patterns observed for *L. minor*. However, this observation may also be the result of differences in exposure duration (96 hr for *L. aequinoctalis* and 168 hr for *L. minor*), because bioavailability relationships may shift under prolonged exposure due to acclimation processes (De Schamphelaere & Janssen, 2004). Together with the observation that the pH extended-algae model resulted in clearly biased predictions relative to observed Ni toxicity for one of the datasets (*L. minor* of Gopalapillai et al., 2013; squares in online supplementary material Figure S2.6, lower panel), it is clear that uncertainty remains regarding the applicability of the (acute) Ni bioavailability models for aquatic plant species.

#### Conclusions on cross-species application of acute Ni bioavailability models

The acute Ni dataset contains toxicity data for several species for which an acute Ni bioavailability model does not exist. For the application of bioavailability models to nonmodel species, it is essential that the validity of the models to predict acute Ni toxicity to nonmodel species is demonstrated (EC, 2018). The use of bioavailability models in SSD approaches for metals assumes that the effects of bioavailability on metal toxicity can be extrapolated between (closely) related species (ECHA, 2008b). In principle, only three bioavailability models, one for each trophic level (i.e., algae, invertebrates, and fish), are needed to normalize the entire acute toxicity dataset covering a diverse set of species to a given physicochemistry and derive an acute site-specific environmental threshold. Cross-species validity of the algae model for different algae species has been demonstrated in Deleebeeck et al. (2009a,b) and Peters et al. (2018). Similarly, it has also been demonstrated that the chronic fish bioavailability model can be used to predict acute Ni toxicity to larval and juvenile fathead minnow (*Pimephales promelas*) and juvenile rainbow trout (*Oncorhynchus mykiss*) accurately (Deleebeeck et al., 2007a). Based on this evidence, it is concluded that the algae model can be used for all algae in the acute Ni effects database, whereas the fish model can be used for all vertebrates in the acute Ni effects database. In this study, we demonstrated that the newly developed pH extended-average invertebrate model can be used to predict acute Ni toxicity for 13 crustacean species, one amphipod, and one annelid, although there were a few datasets for which the prediction performance (based on MPS) was rather low. However, given that the prediction performance varied between different datasets of the same species and the FA was high across all datasets, this study supports the use of the pH extended-average invertebrate model for all invertebrate species. For plant species, the demonstration of cross-species applicability of acute Ni bioavailability models was not unambiguous. However, based on the observation that the pH extended-average invertebrate model performed clearly better than the pH extended-algae model for one of the datasets, it is proposed to use the pH extended-average invertebrate model for evaluating toxicity thresholds of aquatic plants until bioavailability effects for plants are further clarified.

### Acute Ni bioavailability normalization approach

#### Characteristics of the acute Ni bioavailability normalization approach

**Figure 4.**
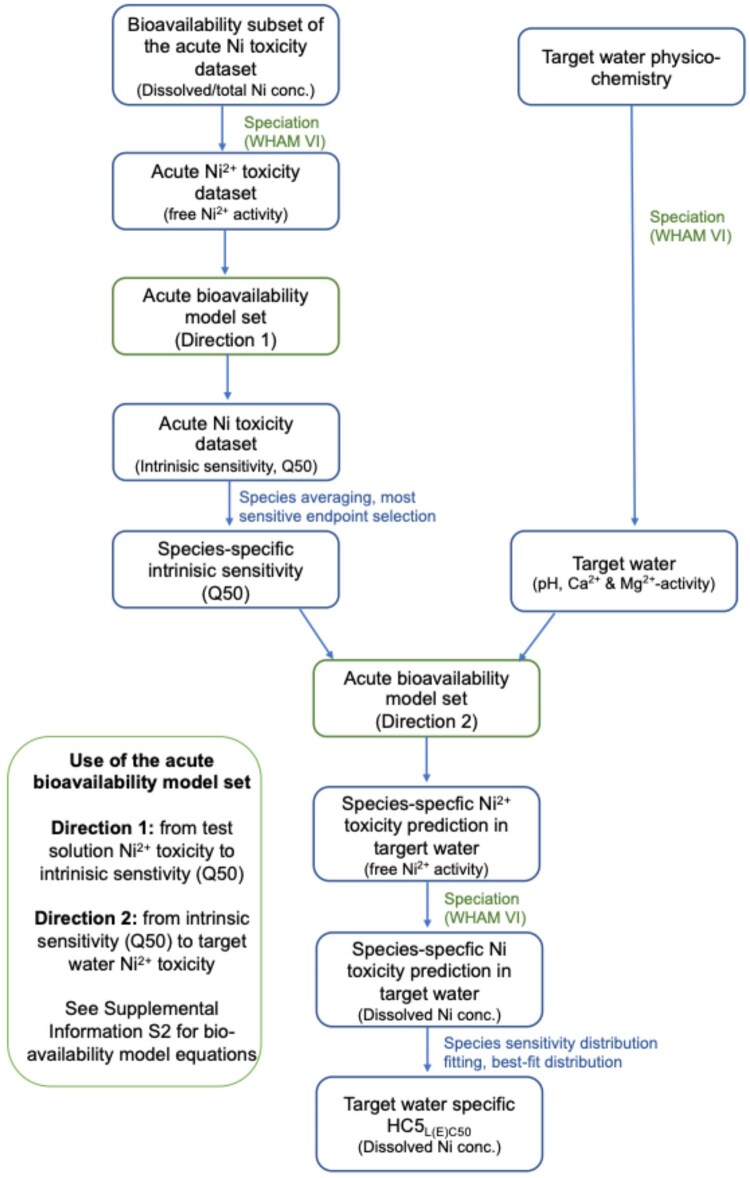
Overview of the acute Ni bioavailability normalization approach. The acute Ni bioavailability normalization approach combines the acute Ni toxicity dataset with the acute Ni bioavailability model set and the species-sensitivity distribution (SSD) approach. The bioavailability normalization procedure starts with translating the acute Ni toxicity database, with Ni toxicity expressed as dissolved or total concentration, to free ion activities in the Windermere Humic Aqueous Model (WHAM) version VI. The free Ni^2+^ toxicity and Mg^2+^ and Ca^2+^ activity resulting from these calculations are used to calculate an intrinsic sensitivity (Q50) for each toxicity data entry separately using the appropriate taxon-specific bioavailability model selected from the acute Ni bioavailability model set (i.e., use of bioavailability models in Direction 1, see online supplementary material S2). After species-averaging and most sensitive endpoint selection based on the intrinsic sensitivity values, the species-specific average intrinsic sensitivity is used for predicting Ni^2+^ toxicity in a target water using the taxon-specific bioavailability model selected from the bioavailability model set and WHAM VI-calculated free Ca^2+^ and Mg^2+^ activities and the pH of the target water (i.e., use of bioavailability models in Direction 2, see see online supplementary material S2). This results in a list of normalized Ni^2+^ toxicities: one for each species per target water, expressed at the free ion activity level. These Ni^2+^ toxicities are then translated back to dissolved Ni concentrations using WHAM VI and the target water-specific physico-chemistry. Finally, a site-specific acute environmental threshold, the HC5_L(E)C50_ (i.e., concentration resulting in at least 50% effect for 5% of the species in the SSD) is calculated.

The acute Ni bioavailability normalization approach combines the acute Ni toxicity dataset (discussed in Section *Acute Ni toxicity dataset*) with the acute bioavailability model set and the SSD approach. Furthermore, this approach removes the variability of the physicochemistry of the test media in the data entries of the acute Ni toxicity dataset and results in the calculation of a site-specific acute environmental threshold represented by the HC5_L(E)C50_. The entire approach is outlined in Figure 4. In addition, an Excel-based tool, including all steps in the bioavailability normalization approach is available in the online supplementary material Supplemental Information S4.

The acute bioavailability model set integrates the fish model (Deleebeeck et al., 2008a), the pH extended-algae model (Deleebeeck et al., 2009a; Nys et al., 2016), and the pH extended-average invertebrate model (this study; see Table 1 for model parameters). Within the bioavailability normalization approach, the fish model is applied to all vertebrates, the pH-extended-algae model to all algae, and the pH extended-average invertebrate model to all invertebrates and plants (see section *Conclusions on cross-species application of acute Ni bioavailability model*). Each of the bioavailability models have been calibrated and validated (on the calibration datasets itself, as well as on independent datasets) over a specific physiochemistry range, that is, the bioavailability model applicability range (see Table 1). As such, the models should only be applied within this range, because predictions outside these ranges are associated with considerable uncertainty. The applicability range of the entire acute Ni bioavailability modeling approach refers to the narrowest window of pH, Ca, and Mg that covers the validated range of all three individual bioavailability models integrated in the acute Ni bioavailability model set. In practice, the acute Ni bioavailability normalization approach can be applied within the physicochemical ranges of pH between 5.7 and 8.7 and hardness between 12 and 290 mg CaCO_3_/L. These ranges cover approximately 70% of the waterbodies within Europe (based on the physicochemistry reported in the EU Physicochemical Database [Metals Environmntal Research Associations, 2020], see also online supplementary material Figure S3.2). The pH and hardness in European freshwaters tend covary, and the pH application boundary of the acute bioavailability set covers the 95th percentile of pH values in European water bodies. As such, extending the acute bioavailability model set to a broader hardness application range would be most efficient in increasing the percentage of waters covered by the acute bioavailability model set.

The SSD approach implemented in the acute Ni bioavailability normalization follows that of the recently updated European chronic Ni bioavailability modeling approach (Peters et al., 2023). This implies that the data aggregation at the species level (within-species-averaging) is done at the intrinsic sensitivity level (Q50), wherea SSD-fitting occurs at the level of the normalized Ni effect concentrations (see Figure 4). The distribution fitting implemented in the acute Ni bioavailability normalization approach selects the best-fitting distribution based on the Anderson-Darling statistic. The implementation of the best-fitting distribution rather than only considering the normal distribution in SSD-fitting is based on the observation that the normal distribution has been reported to fail in fitting ecotoxicity data on several occasions (e.g., Newman et al., 2000; see also next section).

#### Application of the acute Ni bioavailability normalization approach on European freshwater scenarios

Site-specific acute environmental threshold for Ni derived with the acute bioavailability normalization approach for the “ecoregion” scenarios are reported in Table 3, whereas the corresponding species sensitivity distributions are visualized in online supplementary material Figure S3.1, Table S3.1. For the ecoregion set, the best-fit site-specific HC5_L(E)C50_ ranged fourfold, between 51.8 and 229 µg Ni/L (Table 3). The Swedish Lake scenario, which represents soft-water conditions at circum-neutral pH and moderate DOC, results in the lowest HC5_L(E)C50_ estimate (i.e., highest Ni bioavailability conditions), whereas the high hardness and high DOC conditions in the Dutch ditches scenario result in the highest HC5_L(E)C50_ estimate (i.e., lowest Ni bioavailability conditions).

**Table 3.** Overview of bioavailability-normalized site-specific acute environmental thresholds^a^ calculated for seven European freshwater scenarios (i.e., the “ecoregions”) using the best-fitting distribution^b^.

Ecoregion	pH	DOC (mg/L)	Hardness (mg CaCO_3_/L)	Best-fit distribution	HC5_L(E)C50_^c^ (µg Ni/L)	Anderson Darling-statistic; *p*-value
**Lake Monate (Italy)**	7.7	2.5	48	Log-normal	66.3 (46.4–96.8)	*A* = 0.34; *p* = 0.42
**Rhine (Netherlands)**	7.8	2.8	217	Gamma	137 (73.1–259)	*A* = 0.46; *p* = 0.76
**Otter (United Kingdom)**	8.1	3.2	165	Gamma	120 (66.8–215)	*A* = 0.44; *p* = 0.72
**Teme (United Kingdom)**	7.6	8.0	160	Gamma	148 (80.9–267)	*A* = 0.46; *p* = 0.73
**Swedish Lake (Sweden)**	6.7	3.8	28	Gamma	52.6 (30.6–90.2)	*A* = 0.25; *p* = 0.75
**Ebro (Spain)**	8.2	3.7	273	Gamma	126 (68.9–224)	*A* = 0.42; *p* = 0.70
**Ditches (Netherlands)**	6.9	12.0	260	Log-normal	233 (156–363)	*A* = 0.38; *p* = 0.35

a Represented by the HC5_L(E)C50_, the acute 5% hazardous concentration (i.e., concentration that results in at least 50% effect for 5% of the species) expressed in µg dissolved Ni/L.

b All distributions have been fitted to bioavailability-normalized log_10_ transformed species-mean L(E)C50-values.

c The median HC5 (i.e., HC5-50) is reported. The 90% confidence interval on the HC5-50 is reported between brackets (HC5-5 to HC5-95).

*Note.* DOC = dissolved organic carbon.

The fit of the normal distribution to the bioavailability normalized acute Ni SSD was always significant for the ecoregion scenarios (*p* > 0.05; see online supplementary material Table S3.3). However, a visual inspection of the fitted SSDs (normal vs. best-fit SSD) for these seven ecoregions indicates that the normal distribution does not fit the log-transformed species mean toxicity data very well in the lower tail of the SSD for most ecoregion scenarios (See online supplementary material Figure S3.1). Hence, the estimate of the HC5_L(E)C50_ using a best-fit distribution approach can be regarded as a more accurate estimate of the acute Ni environmental threshold than that of the normal distribution.

**Figure 5.**
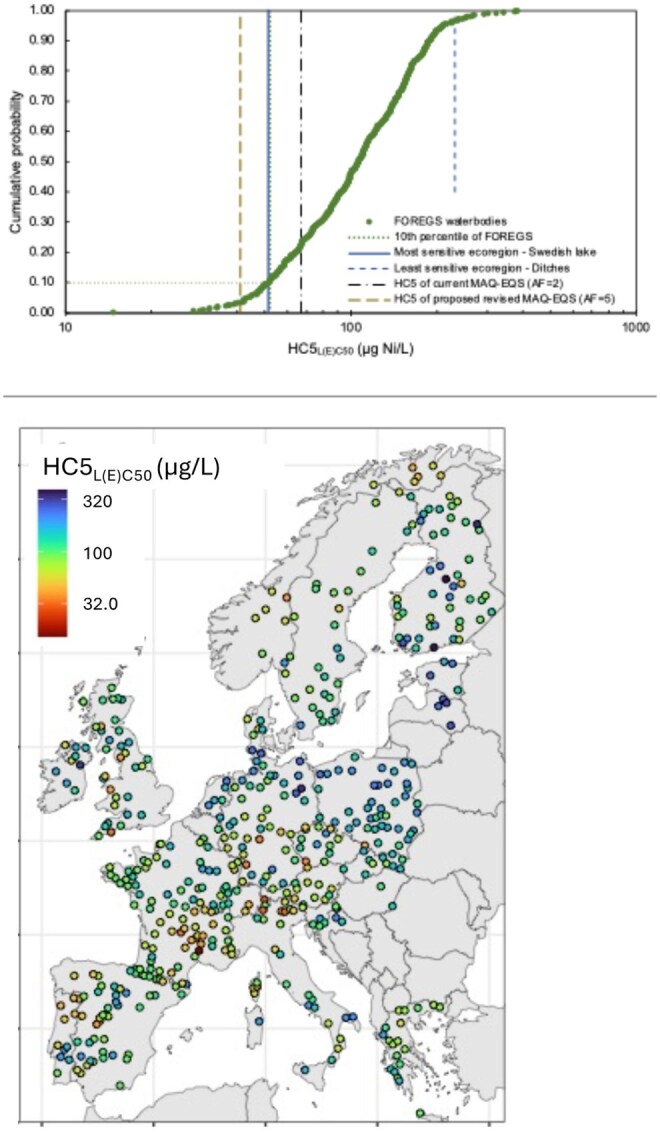
Distribution of site-specific acute environmental thresholds, expressed as the HC5_L(E)C50_ (concentration [µg dissolved Ni/L] that results in at least 50% effect for 5% of the species in the acute Ni toxicity database) in European waterbodies (based on the database of the Forum of the European Geological Surveys [FOREGS]; Salminen et al., 2005) represented as cumulative distribution (upper panel) and geographical distribution (right panel). Only waterbodies with physicochemistry within the bioavailability model ranges (pH: 5.7–8.7; *N* = 488) have been considered. The range of site-specific HC5_L(E)C50_ for the “ecoregions” is indicated using vertical lines showing the HC5_L(E)C50_ of the most sensitive ecoregion (full line) and the least sensitive ecoregion (dashed line). The tenth percentile of HC5_L(E)C50_ is indicated by the horizontal dotted line. All HC5_L(E)C50_ have been calculated based on the best-fit distribution and represent median 5% hazardous concentrations. For comparison the HC5_L(E)C50_ underlying the current Maximum Allowable Concentration-Environmental Quality Standard (MAC-EQS; EC, 2013) including an assessment factor (AF) of 2 and the proposed revised MAC-EQS (EC, 2022) including an AF of 5 are indicated by the brown dashed and black dashed dotted line, respectively. All HC5_L(E)C50_ are reported in online supplementary material Supplemental Information S5.

Within the surface water set representative of European waterbodies (FOREGS), site-specific acute environmental thresholds range between 15.2 µg Ni/L (pH 8.7, DOC 0.5 mg/L, and hardness 37 mg CaCO_3_/L) and 388 µg Ni/L (pH 6.6, DOC 72 mg/L, and hardness 20 mg CaCO_3_/L; Figure 5; see online supplementary material Supplemental Information S5). The lower tenth percentile of the distribution of site-specific HC5_L(E)C50_ (51.9 µg Ni/L) is very similar to the HC5_L(E)C50_ of the most sensitive ecoregion. The lower tenth percentile of the distribution of HC5_L(E)C50_ of the FOREGS waters are waterbodies characterized by soft water conditions (hardness between 12 and 60 mg CaCO_3_/L), low DOC (DOC < 2.5 mg/L), and/or high pH conditions (pH ≥ 8.2; see online supplementary material Figure S3.3), conditions that are mainly encountered in Europe in mountainous regions (e.g., the Alps) or Norway (Figure 5, right panel). These same conditions have also been identified to represent conditions with high Ni bioavailability relative to the chronic environmental threshold derivation (Peters et al., 2023).

The current MAC-EQS (34 µg dissolved Ni/L; assessment factor of 2; EC, 2013) and proposed MAC-EQS (8.2 µg dissolved Ni/L; assessment factor of 5; EC, 2022) for compliance assessment under the EU WFD have also been derived using a species-sensitivity distribution approach. However, none of the MAC-EQS derivations included a bioavailability correction. The corresponding HC5_L(E)C50_ of the current MAC-EQS and proposed MAC-EQS are equal to 67 and 41 µg dissolved Ni/L, respectively (EC, 2008a; EC, 2022). When compared with the calculated HC5_L(C)50_ in this study for the ecoregion scenarios and the broader European surface water set, the current and proposed MAC-EQS can be over and underprotective depending on the local bioavailability situation (See Figure 5). The latter stresses the importance of taking bioavailability into consideration in environmental quality standard derivation for metals. The SCHEER strongly recommended to derive the MAC-EQS for Ni using a bioavailability normalization approach (SCHEER, 2022). Our study and its associated bioavailability tool provide a robust method to implement bioavailability in the derivation of the MAC-EQS. In addition, it allows the integration of environmental relevance in compliance evaluations under the Water Framework Directive by the consideration of local bioavailability conditions.

## Conclusion

In this study, we developed a bioavailability-based effects assessment method for Ni to derive acute freshwater environmental threshold levels for use in the relevant EU regulations which was, to our knowledge, not yet available. The acute environmental threshold approach includes a high-quality ecotoxicity database covering 63 different freshwater species and is combined with an acute bioavailability model set to allow the derivation of site-specific acute environmental thresholds for Ni using a species sensitivity distribution approach. The underlying bioavailability models for invertebrates (developed in the study), algae (Deleebeeck et al., 2009a), and fish (Deleebeeck et al., 2007a) have been extensively validated with (independent) data from several species. As such, the bioavailability models represent a robust approach for incorporating bioavailability considerations in acute environmental threshold derivation, although some uncertainties remain related to the Ni bioavailability relationships for plant species. The applicability ranges of the acute Ni bioavailability normalization approach (pH 5.7–8.7 and hardness 12–290 mg CaCO_3_/L) are estimated to be valid for approximately 70% of European freshwaters.

The proposed acute Ni bioavailability normalization approach and the associated bioavailability normalization tool available in online supplementary material Supplemental Information S4 can serve as the basis for deriving acute bioavailability-based environmental threshold values for Ni in freshwater, such as the MAC-EQS under the WFD and the PNEC_intermittent_ under REACH. As such, the approach can increase the scientific relevance of compliance evaluation relative to acute environmental threshold values for Ni in the EU.

## Supplementary Material

vgae071_Supplementary_Data

## Data Availability

All data used in our study are published. Data and calculations tools for the acute Ni bioavailability normalization approach are included in the Supporting Information. Data not included in Supporting Information can be shared on request.
