# Supplementary material for: Development of a bioavailability-based acute effects assessment method for nickel
Source: Environ Toxicol Chem. 2025 Jan 6;44(3):841–55. doi: 10.1093/etojnl/vgae071 (PMC11864204; doi:10.1093/etojnl/vgae071)
Supplement: vgae071_Supplementary_Data [file vgae071_supplementary_data.zip › vgae071_Supplementary_Data/S1-S3_Acute Ni BLM paper.pdf]

Supplemental Information to "***Development of a bioavailability-based acute effects assessment method for Ni " – Nys et al. 2025***

Supplemental Information

S1: Speciation assumption details……………………………………………………………………………2

S2: Validation of the (pH extended)-average invertebrate model for invertebrate and plant species – detailed analysis………………………………………………………………………………………………3

S3: Overview of detailed physico-chemistry of ecoregion scenarios used for calculating site specific $HC5_{L(E)C50}$ and fitted species sensitivity distributions………….………………….   ………………….…20.

SI4: Acute Ni bioavailability normalization tool (including acute Ni freshwater toxicity database + overview of non-retained data)

(Separate zip-file)

SI5: Reporting all normalized site-specific acute environmental threshold ($HC5_{L(E)C50}$ values) and corresponding physico-chemistry input for the European surface water set

(Separate excel-file)

S1: Speciation assumption details

For all bioavailability modelling, speciation of Ni^{2+} and other ions was calculated with the software package WHAM (Windermere Humic Aqueuos Model) VI (Tipping, 1998; Natural Environment Research Council, 2001). The default stability constants of Ni for inorganic complexes were adapted to those of NIST (*National Institute of Standards and Technology*). Adapted and default parameters are shown in Table S1. 1. For Dissolved Organic Matter (DOM) from natural sources (e.g. tap water, surface water), we assumed that 40% of the DOM is reactive and behaves as isolated fulvic acid, with the remaining 60% of the DOM content not being reactive. Additionally, it was assumed that DOM contains 50% carbon on a weight basis (Ritchie & Perdue, 2003). Accordingly, the measured Dissolved Organic Carbon (DOC) content (mg C/L) for natural sources was multiplied by a factor of 0.8 to obtain the amount of FA (mg FA/L) to be used as the modeling input. For synthetic waters for which no DOC concentration was reported (and that are expected to contain less than 1 mg/L DOC), concentrations of FA were set to zero, since background concentrations of DOC (<1 mg/L) were shown to not substantially influence Ni speciation (De Schamphelaere et al. 2006). If DOC was reported, speciation calculations used the reported DOC concentrations. Furthermore, an optimized Ni-fulvic acid binding constants (log $K_{Ni\text{-}FA}$) of 1.75 was used (Van Laer et al. 2006). Finally, we assumed that activities of the metal cations Fe^{3+} and Al^{3+} - both competing with Ni^{2+} for binding sites on FA – were controlled by their colloidal $Fe(OH)_3$ and $Al(OH)_3$ precipitates as explained in Cheng et al. (2005).

Table S1. 1 Default and adapted stability constants of Ni for inorganic complexes

Parameter	Default stability constant in WHAM VI (log K)	Adapted stability constant (log K)
$K = [NiCO_3]/\{[Ni^{2+}].[CO_3^{2-}]\}$	5.78	4.57
$K = [NiHCO_3^+]/\{[Ni^{2+}].[H^+].[CO_3^{2-}]\}$	13.41	12.42

S2 Validation of the (pH extended)-average invertebrate model for invertebrate and plant species – detailed analysis

S2.1 Datasets used for cross-species validation

Peer-reviewed and available grey literature was searched for studies that evaluated bioavailability effects on acute Ni toxicity to invertebrates and plants. Data was only included in the present study if acute Ni toxicity for a species was tested in at least two test waters with differing physico-chemistry in the same study. Bioavailability effects data from 12 invertebrate studies covering, in total, 15 species (13 crustacean, 1 amphipod and 1 annelid) and 3 plants studies (2 species) were identified. An overview of the different datasets selected for evaluating the predictive capacities of the acute invertebrate Ni bioavailability models for invertebrates and plants is given in Table 1.

Each dataset used for bioavailability modelling is discussed in more detail below:

Ceriodaphnia dubia: De Schamphelaere et al. (2006) tested acute (48h) Ni toxicity to *C. dubia* in 6 different natural waters. Water chemistry and 48h-$LC50_{Nidiss}$ was taken from their Annex 3.

Keithly et al. (2004) reported on the effect of hardness on acute Ni toxicity to *C. dubia*. They tested in total 4 hardness levels ranging from 50 to 253 mg $CaCO_3$/L. Water chemistry and 48h-$LC50_{Nidiss}$ was taken from De Schamphelaere et al. 2006 (Annex 9).

Parametrix (2004) reported on the effect of an increase in pH on acute Ni toxicity to *C. dubia.* They tested 4 pH level (pH 6, 7, 8 and 9), which were buffered with MOPS. Additionally, for pH 8 and 9 they also tested Ni toxicity without adding MOPS-buffer. Water chemistry and 48h-$LC50_{Nidiss}$ was taken from De Schamphelaere et al. 2006 (Annex 10).

Daphnia magna: Deleebeeck et al. (2008) reported on the effect of Ca, Mg, Na and pH (both controlled with $NaHCO_3$ and MOPS) on acute (48h) Ni toxicity. These data were used by the authors to develop the *D. magna* acute Ni model. Additionally, they validated their model in 8 different natural waters (16 individual tests). In total, 52 synthetic and natural waters were tested. Water chemistry and 48h-$LC50_{Nidiss}$ was taken from their Table 1 and 2.

Chapman et al. (1980) tested the effect of hardness on acute Ni toxicity to *D. magna.* In total 5 waters were tested including 4 different hardness levels ranging from 50 to 250 mg $CaCO_3$/L. Water chemistry and 48h-$LC50_{Nidiss}$ was taken from De Schamphelaere et al. 2006 (Annex 6).

Mano & Shinohara (2020) tested acute (48h) Ni toxicity in 5 Japanese natural waters. Each natural water was tested at two pH levels (i.e. either pH 6.8 and 7.4 or pH 7.4 and 8.2). Water chemistry was taken from their Table 2 and 48h-LC50 from their Table 3.

Daphnia pulex: Acute Ni toxicity data for *D. pulex* was obtained from two separate studies. First, Kozlova et al. (2009) tested acute (48h) Ni toxicity to *D. pulex* in 44 synthetic waters in 8 different test series

differing in Ca, Mg, K, Na and DOC concentration, pH and DOC origin. Water chemistry and 48h-$LC50_{Nidiss}$s were taken from their Table 1.

Second, Leonard and Wood (2013) reported on acute (48h) Ni toxicity to *D. pulex* at two levels of water hardness levels. Water chemistry for modelling was taken from their Table 1. The 48h-$LC50_{Nidiss}$s were taken from their Table 2.

<u>*Hyalella azteca*</u>: Borgmann et al. (2005) reported on acute (7d) Ni toxicity to *H. azteca* at two levels of water hardness. Water chemistry for modelling was taken from their materials and methods. The measured 7d-$LC50_{Nidiss}$ were taken from their Table 3.

Schroeder et al. (2010) reported on acute (7d) and chronic (28d) Ni toxicity to *H. azteca* in 28 different test waters. Test waters varied in hardness (Ca and/or Mg), pH and alkalinity. Water chemistry for modelling was taken from their Table 1. 7d-$LC50_{Nidiss}$ were taken from their Table 3. It should be noted that Schroeder et al. (2010) reported that nominal Ni concentrations were not confirmed for all test concentrations by measurements, but the authors reported that for the test concentrations that were measured Ni concentration on the day of solution preparation was on average 93% of the nominal addition

Schubauer-Berigan et al. (1993) reported on acute (96h) Ni toxicity to *H. azteca* in very hard reconstituted water at three pH levels (6.3, 7.3 & 8.3). The measured 96h-$LC50_{Nidiss}$ were taken from their Table 2. Bioavailability modelling was conducted based on the nominal concentration of very hard reconstituted water (USEPA 2002) in combination with measured pH values as reported in Table 1.

<u>*Lumbriculus variegatus*</u>: Leonard and Wood (2013) reported on acute (96h) Ni toxicity to *L. variegatus* at two levels of water hardness. Water chemistry for bioavailability modelling was taken from their Table 1. The 96h-$LC50_{Nidiss}$ were taken from their Table 2.

Schubauer-Berigan et al. (1993) reported on acute (96h) Ni toxicity to *L. variegatus* in very hard reconstituted water at three pH levels (6.3, 7.3 & 8.3). The measured 96h-$LC50_{Ni}$ were taken from their Table 2. Bioavailability modelling was conducted based on the nominal concentration of very hard reconstituted water (USEPA 2002) in combination with measured pH values as reported in Table 1.

<u>Non-model Crustacean dataset:</u> Deleebeeck et al. (2007) tested acute (48h) Ni toxicity for 10 Crustacean species in 11 experiments (22 toxicity datapoints in total: *Ceriodaphnia quadrangula (2 exp.), Daphnia longispina, Alona affinis, Camptocercus lilljeborgi, Ceriodaphnia pulchella, Chydorus ovalis, Simocephalus vetulus, Peracantha truncata, Simocephalus serrulatus* and *Bosmina corregoni*. These Crustaceans were isolated from either a soft field water or a hard field water. Acute Ni toxicity for each species was evaluated at two hardness concentrations (i.e. either soft vs. moderately hard water or moderately hard vs. hard water). Water chemistry for bioavailability modelling was taken from their Table 2. The 48h-$LC50_{Nidiss}$ were taken from their Table 3.

It should be noted that Leonard & Wood (2013) also tested acute (96h) Ni toxicity to *Chironomus riparius* at two hardness levels. However, LC50s could not be accurately established for this species. Therefore, this data was not considered for this exercise.

Table S2. 1 Overview of invertebrate data used for validating the acute Ni bioavailability models

Species	Reference	Number of datapoints	Test duration	Bioavailability parameter evaluated + range of main physico-chemistry parameters
**Invertebrates**				
Ceriodaphnia dubia	De Schamphelaere et al. 2006	6	48h	Natural water (pH: 6.4-8.0; DOC:3.1-23.6 mg/L; hardness: 15.0-218 mg $CaCO_3$/L)
Ceriodaphnia dubia	Keithly et al. 2004	4	48h	Hardness (pH: 7.6-7.8; DOC: 0 mg/L; hardness: 50-252 mg $CaCO_3$/L)
Ceriodaphnia dubia	Parametrix 2004	6	48h	pH (pH: 6.3-8.9; DOC= 0 mg/L; hardness: 93-105 mg $CaCO_3$/L)
Daphnia magna	Deleebeeck et al. 2008	52	48h	Ca, Mg, Na and pH, natural water (pH: 5.7-8.1; DOC:0.0-25.8 mg/L; hardness: 13.2-475 mg $CaCO_3$/L)
Daphnia magna	Chapman et al. 1980	5	48h	Hardness (pH: 7.7-8.3 DOC: 0 mg/L; hardness: 49-199 mg $CaCO_3$/L)
Daphnia magna	Mano & Shinohara 2020	10	48h	Natural water + pH (pH: 6.8-8.2 DOC: 0.5-2.6 mg/L; hardness: 21-158 mg $CaCO_3$/L)
Daphnia pulex	Kozlova et al. 2009	44	48h	Ca, Mg, K, Na, pH and DOC concentration and origin: (pH: 5.6-8.3 DOC: 0-20.5 mg/L; hardness: 16-161 mg $CaCO_3$/L)
Daphnia pulex	Leonard & Wood 2013	2	48h	Hardness (pH: 7.2-7.8 DOC: 1.2-2.3 mg/L; hardness: 51-141 mg $CaCO_3$/L)
Hyalella azteca	Borgmann et al. 2005	2	7d	Hardness (pH: 7.4-8.4 DOC: 0.3-1.1 mg/L; hardness: 18-123 mg $CaCO_3$/L)
Hyalella azteca	Schroeder et al. 2010	28	7d	Hardness, pH, alkalinity (pH: 6.4-8.9 DOC: 0.2-0.7 mg/L; hardness: 10-300 mg $CaCO_3$/L)
Hyalella azteca	Schubauer-Berignan et al. 1993	3	96h	pH (pH: 6.2-8.4 DOC: 0 mg/L; hardness: 339 mg $CaCO_3$/L)
Lumbriculus variegatus	Leonard & Wood 2013	2	96h	Hardness (pH: 7.2-8.8 DOC: 1.2-2.3 mg/L; hardness: 51-141 mg $CaCO_3$/L)
Lumbriculus variegatus	Schubauer-Berignan et al. 1993	3	96h	pH (pH: 6.5-8.3 DOC: 0 mg/L; hardness: 339 mg $CaCO_3$/L)
Crustacean dataset a	Deleebeeck et al. 2007	2 (10 species)	48h	Hardness (pH: 7.2 DOC: 0 mg/L; hardness: 6.3-43.4 mg $CaCO_3$/L)
**Plants**				
Lemna minor	Schlekat et al. 2010	6	7d	Natural water (pH: 6.9-8.3; DOC: 0.7-7.1 mg/L; hardness: 35.0-237 mg $CaCO_3$/L)
Lemna minor	Gopalapillai et al. 2013	50	7d	Hardness, Na, K (pH: 8.1-8.3; DOC: 0.5 mg/L; hardness: 149-1099 mg $CaCO_3$/L)
Lemna minor	Nys et al. 2016	2	7d	pH (pH: 8.2-8.7; DOC: 0 mg/L; hardness: 175 mg $CaCO_3$/L)
Lemna aequinoctalis	Peters et al. 2018	5	96h	Natural water: (pH: 6.7-8.4; DOC: 0.5-10 mg/L; hardness: 1.7-58 mg $CaCO_3$/L)

a In the study of Deleebeeck et al (2007), acute Ni toxicity was evaluated for the following 10 crustacean species: *Ceriodaphnia quadrangula, Daphnia longispina, Alona affinis, Camptocercus lilljeborgi, Ceriodaphnia pulchella, Chydorus ovalis, Simocephalus vetulus, Peracantha truncata, Simocephalus serrulatus* and *Bosmina corregoni*

<u>*Lemna minor*</u>*:* Schlekat et al. (2010) reported on chronic (7d) Ni toxicity to *L. minor* in 5 natural waters and a synthetic medium. Water chemistry was taken from their Table A-4 from the supplementary material. 7d-$EC50_{Nidiss}$ were taken from their Table 3.
Gopalapillai et al. 2013 reported on chronic (7d) Ni toxicity to *L. minor* in 25 artificial media, which varied in concentrations of Ca, Mg, Na and K. Water chemistry was partly (only pH and major ion varied has been reported) reported in their Table 1. Other ions were assumed to be the same as in the control solution. 7d-$EC50_{Nidiss}$ were taken from the supplemental data (Table S-1). All bioavailability modeling was based on the frond count endpoint, while the root length-data was not considered.
Nys et al. (2016) reported on the effect of high pH (pH>8.2) on 7d-Ni toxicity to *L. minor* in 2 artificial medias. Ni toxicity (growth rate) was evaluated at pH 8.2 and pH 8.7, and 7d-$EC50_{Nidiss}$ values were taken from their Table 2. Water chemistry was taken from their supplemental data.

<u>*Lemnae aequinoctalis*</u>: Peters et al. (2018) reported on 96h-Ni toxicity to *L. aequinoctalis* in 5 natural waters representing the range of physico-chemistry parameters (pH, Ca and Mg) in Australian waters. Water chemistry and effect concentrations were taken from their supplementary material.

S2.2 Acute Ni bioavailability models for invertebrates and algae

The (acute) Ni bioavailability models are explained in more detail in the main manuscript. Specific model equations are given in Table S2.2 for the (pH extended-)-average invertebrate models and Table S2.3 for the pH extended-algae model.

Table S2. 2 Overview of model equations depending on the considered pH range for the ‘pH-extended average invertebrate model’

pH range	Model	Model equation	Intrinsic sensitivity calculation
5.7-8.0	‘Basic average invertebrate model’	$LC50_{Ni^{2+},i,predicted} = 10^{-(Q50_{Ni2+,species\ k}+S_{pH,invertebrate,pH\leq 8.0}\times(pH_i))} \times (1+K_{CaBL,invertebrate}\{Ca^{2+}\}_i + K_{MgBL,invertebrate}\{Mg^{2+}\}_i)$	$Q50_{Ni2+,species\ k} = -\log\left(\frac{LC50_{Ni^{2+},observed\ species\ k,i}}{1+K_{CaBL,invertebrate}\{Ca^{2+}\}_i + K_{MgBL,invertebrate}\{Mg^{2+}\}_i}\right) - S_{pH,invertebrate,pH\leq 8.0}\times(pH_i)$
8.0-8.9	High pH extension	$LC50^{pred\ high\ pH\ model\ j}_{Ni^{2+},i} = 10^{-(Q50_{Ni2+,species\ k}+S_{pH,invertebrate,pH>8.0}\times(pH_i-8.0))} \times (1+K_{CaBL,invertebrate}\{Ca^{2+}\}_i + K_{MgBL,invertebrate}\{Mg^{2+}\}_i)$	$Q50_{Ni2+,species\ k} = -\log\left(\frac{LC50_{Ni^{2+},observed\ species\ k,i'}}{1+K_{CaBL,invertebrate}\{Ca^{2+}\}_i+K_{MgBL,invertebraete}\{Mg^{2+}\}_i}\right) - S_{pH,invertebrate,pH>8.0}\times(pH_i-8.0)$

Where $Q50_{Ni2+,\ species\ k}$ is the intrinsic sensitivities for species k (mol/L). $K_{CaBL,invertebrate}$ is the stability constant for binding of Ca^{2+} to the Ni biotic ligand of the average invertebrate model *j (*L/mol; log K_{CaBL}=3.80). $K_{MgBL,invertebrate}$ is the stability constant for binding of Mg^{2+} to the Ni biotic ligand of the average invertebrate model *j* *(*L/mol; log K_{MgBL}=3.32). S_{pH} is the slope of the log-linear relationship between pH and Ni^{2+} activity, $S_{pH,invertebrate,\ pH\leq 8.0}$ denotes the S_{pH}-parameter valid for pH up to 8.0 ($S_{pH,invertebrate,\ pH\leq 8.0}$=0), and $S_{pH,invertebrate,\ pH>8.0}$ denotes the S_{pH}-parameter valid in the pH range 8.0 and 8.9 ($S_{pH,invertebrate,\ pH>8.0}$=1.01; Present study) . $\{Mg^{2+}\}_i$, and $(Ca^{2+})_i$ are the chemical activities (M) of $Mg^{2+}$and Ca^{2+} in a test solution or target water *i* (mol/L), respectively. pH_i is the pH of the considered test solution or target water *i.* $LC50_{Ni2+,observed\ speciesk,i}$ is the observed acute Ni^{2+} toxicity in test solution *i* for species *k.*

Table S2. 3 Overview of model equations depending on the considered pH range for the ‘pH-extended algae model’

pH range	Model	Model equation	Intrinsic sensitivity calculation
5.7-8.2	‘Basic algae model’	$EC50_{Ni^{2+},i,predicted} = 10^{-(S_{pH,Algae}\cdot pH_i+Q50_{Algae})}\cdot(1+K_{MgBL,Algae}\{Mg^{2+}\}_i)$	$Q50_{Ni2+,species\ k} = -\log\left(\frac{EC50_{Ni^{2+},observed\ species\ k,i}}{1+K_{MgBL,algae}\{Mg^{2+}\}_i}\right) - S_{pH,algael}\cdot pH_i$
8.2-8.7	High pH extension	<u>Two-step approach:</u><br>First normalize up to pH 8.2 using<br>$EC50_{Ni^{2+},i,predicted\ at\ pH\ 8.2} = 10^{-(S_{pH,Algae}\cdot pH_i+Q50_{Algae})}\cdot(1+K_{MgBL,Algae}\{Mg^{2+}\}_i)$<br>Second normalize to pH>8.2 using<br>$EC50^{pred\ high\ pH\ model\ j}_{Ni^{2+},at\ pH>8.2} = 10^{(\log EC50_{Ni^{2+},i,predicted\ at\ pH\ 8.2} - S_{pH,high\ pH\ model\ j}\times(pH_i-8.2))}$	<u>Two-step approach:</u><br>First normalize Ni-toxicity down to pH=8.2 using the pH extension<br>$EC50_{Ni^{2+},at\ pH=8.2} = 10^{(\log EC50_{Ni^{2+},i,observed\ at\ p>8.2} + S_{pH,high\ pH\ algae/plant}\times(pH_i-8.2))}$<br>Second calculate Q using the basic algae model<br>$Q50_{Ni2+,species\ k} = -\log\left(\frac{EC50_{Ni^{2+},at\ pH=8.2}}{1+K_{MgBL,algae}\{Mg^{2+}\}_i}\right) - S_{pH,algae}\cdot 8.2$

Where $Q50_{Ni2+,\ species\ k}$ is the intrinsic sensitivities for species k (mol/L). $K_{MgBL,algae\ j}$ is the stability constant for binding of Mg^{2+} to the Ni biotic ligand of the algae model *j* *(*L/mol; log K_{MgBL}=3.32). $S_{pH,algae}$ is the slope of the log-linear relationship between pH and Ni^{2+} activity for pH up to 8.2 (S_{pH}=0.143; Deleebeeck et al. 2009) $S_{pH,high\ pHalgae/plant}$ is the slope of the log-linear relationship between pH and Ni^{2+} activity of the algae/plant model in the pH range 8.2 and 8.7 (S_{pH}=0.906; Nys et al. 2016) . $\{Mg^{2+}\}_i$ is the chemical activities (M) of Mg^{2+} in a test solution or target water *i* (mol/L), respectively. pH_i is the pH of the considered test solution or target water *i.* $EC50_{Ni2+,observed\ speciesk,i}$ is the observed acute Ni^{2+} toxicity in test solution *i* for species *k.*

S2.3 Results of the bioavailability modeling for invertebrate species

Table S2. 4 Intrinsic sensitivities (Q50) and prediction errors for the species-specific model[a] and the average invertebrate model for different invertebrate species.

Species	Reference[a]	Test duration	Toxicity modifying factor [b] (# of samples)	Species-specific model		Average invertebrate model		Remark
				Intrinsic sensitivity	Prediction error[e]	Intrinsic sensitivity	Prediction error[e]	
Ceriodaphnia dubia	[1]	48h	Natural water (n=6)	6.85±0.22	1.28±0.32	7.12±0.27	1.51±0.42	
	[2]	48h	Hardness (n=4)	6.13±0.11	1.24±0.08	6.42±0.08	1.18±0.02	
	[3]	48h	pH (n=4 [pH≤8.3]; n=6 [pH≤8.9])	6.10±0.21 Only pH≤8.3 (n=4)	1.43±0.53	6.27±0.22 All pH (n=6)	1.52±0.37	
Daphnia magna	[4]	48h	Ca, Mg, Na and pH, natural water (n=52)	4.65±0.13[a]	1.30±0.22	5.01±0.20[a]	1.53±0.32	sensitivities were calculated based on all waters, with the exception of Ca>3mM and Mg>3mM treatments (i.e. 48 test media in total).
	[5]	48h	Hardness, pH (n=5)	4.99±0.20	1.40±0.50	5.27±0.20[c]	1.44±0.42	
	[6]	48h	Natural water + pH (n=10)	4.89±0.19	1.48±0.30	5.17±0.11	1.22±0.18	
Daphnia pulex	[7]	48h	Ca, Mg, K, Na, pH, DOC (n=44)	4.69±0.19	1.36±0.37	4.95±0.19	1.40±0.54	
	[8]	48h	Hardness (n=2)	5.46[d]	1.18	5.80[d]	1.34	Calibrated on the soft water only
Hyalella azteca	[9]	7d	Hardness (n=2)	NA	NA	6.23[d]	1.39	Calibrated on the soft water only
	[10]	7d	Hardness, pH, alkalinity (n =21)	NA	NA	6.17±0.26	1.59±0.40	intrinsic sensitivity calculation is based on all water with Ca≦3 mM (n=21).
	[11]	96h	pH (n=3)	NA	NA	5.82±0.08	1.16±0.10	
Lumbriculus variegatus	[8]	96h	Hardness (n=2)	NA	NA	4.74[d]	1.20	Calibrated on the low hardness water.
	[11]	96h	pH (n=3)	NA	NA	4.25±0.25	1.55±0.35	
10 Crustacean species	[12]	48h	Hardness (n=2)	NA	NA	See Table S2.5	1.45 ±0.39 (see Table S2.5)	

[a] References: [1]De Schamphelaere et al. 2006; [2] Keithly et al. 2004; [3] Parametrix 2004; [4] Deleebeeck et al. 2008; [5] Chapman et al. 1980; [6] Mano & Shinohara 2020; [7] Kozlova et al. 2009; [8] Leonard & Wood 2013; [9] Borgmann et al. 2005; [10] Schroeder et al. 2010; [11] Schubauer-Berignan et al. 1993; [12] Deleebeeck et al. 2007b

[b] Potential toxicity modifying factors (TMF) considered in the dataset. Natural water, indicates that a set of natural waters with different physicochemical conditions has been evaluated. Note that only waters within the applicability ranges of the considered bioavailability model has been considered for calculating performance scores.

[c] For model-species the species-specific model indicates that the *C. dubia* model was used for all *C. dubia*-datasets, the *D. magna* model was used for all *D. magna*-datasets *and* the *D. pulex* model was used for all *D. pulex*-datasets. Species-specific models are only calibrated & applied on toxicity data with pH≤8.3, which represents roughly the applicability ranges of the species-specific models (see Table 1, main paper).

[d] For datasets with only two datapoints, the intrinsic sensitivity of the average invertebrate model was calibrated on the water with lowest hardness, and acute Ni toxicity was predicted in the other test medium.

[e] Average prediction error ± standard deviation is reported

h=hours, d= days, DOC= dissolved organic carbon, NA= not applicable

Table S2. 5 Species-specific intrinsic sensitivities (Q50)[a] for 10 crustacean species[b] for the average invertebrate model

	Average invertebrate model $Q50_{Ni2+}$ (log mol/L)[a]
Ceriodaphnia quadrangula	5.48
Daphnia longispina	5.4
Alona affinis	4.60
Camptocercus lilljeborgi	5.35
Ceriodaphnia pulchella	5.15
Chydorus ovalis	4.56
Simocephalus vetulus	5.39
Ceriodaphnia quadrangula	5.93
Peracantha truncata	4.64
Simocephalus serrulatus	4.92
Bosmina corregoni	5.33

a Toxicity was tested either in soft (6.3 mg CaCO3/L) vs. moderately hard water (16.3 mg CaCO3/L) or moderately hard (16.3 mg CaCO3/L) vs. hard water (43.4 mg CaCO3/L). Intrinsic sensitivity was calibrated based on the moderately hard water.

b Toxicity data from Deleebeeck et al. (2007)

S2.2.2 Additional information on the prediction performance of the average invertebrate model

Table S2. 6 Performance[a] of the average invertebrate model for the crustacean species in the dataset of Deleebeeck et al. (2007).

	Prediction in	Performance scores				Residual scores for Ca and Mg		Prediction error
		r^2	FA	Tot RS	MPS	RS Ca	RS Mg	
Ceriodaphnia quadrangula	Soft water	0.00	1.00	0.59	0.53	0.64	0.53	1.40
Daphnia longispina		0.00	1.00	0.45	0.48	0.52	0.39	1.60
Alona affinis		1.00	1.00	0.97	0.99	0.97	0.97	1.02
Camptocercus lilljeborgi		0.93	1.00	0.83	0.92	0.85	0.80	1.14
Ceriodaphnia pulchella		0.00	1.00	0.49	0.50	0.56	0.43	1.54
Chydorus ovalis		0.93	1.00	0.57	0.83	0.63	0.51	1.42
Simocephalus vetulus		0.53	1.00	0.39	0.64	0.46	0.32	1.72
Ceriodaphnia quadrangula	Hard water	0.99	1.00	0.97	0.99	0.98	0.97	1.02
Peracantha truncata		0.22	1.00	0.82	0.68	0.85	0.80	1.14
Simocephalus serrulatus		0.34	1.00	0.47	0.60	0.53	0.40	1.58
Bosmina corregoni		0.00	0.50	0.19	0.23	0.25	0.13	2.38

[a] Performance scores indicate the capacity of the average invertebrate model to predict acute Ni toxicity in either the soft (6.3 mg CaCO3/L) or hard water (43.4 mg CaCO3/L) when the intrinsic sensitivity was calibrated based on the moderately hard water (16.3 mg CaCO3/L).

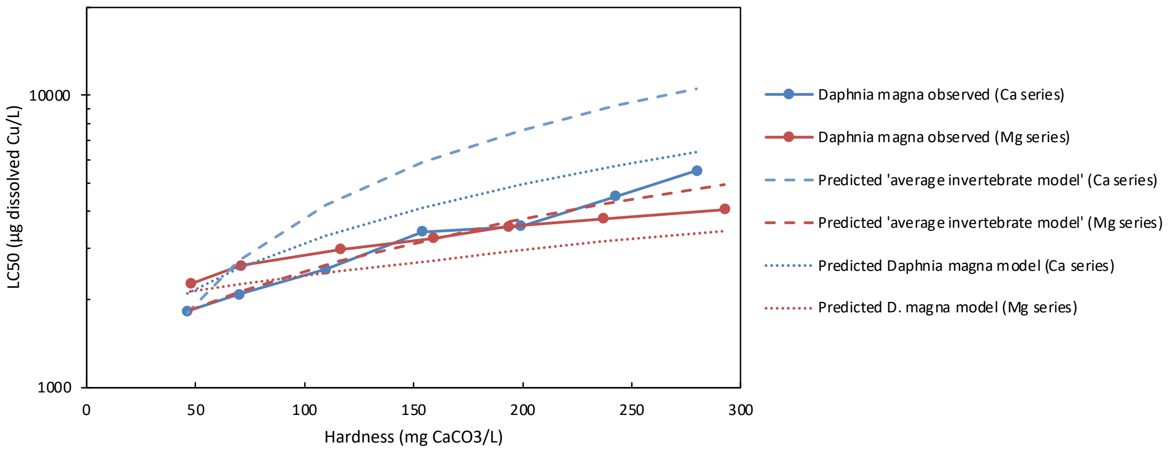

Figure S2. 1 Overview of observed effect (full lines and symbols) of hardness on acute Ni toxicity (LC50; µg dissolved Ni/L) to *Daphnia magna* in the dataset of Deleebeeck et al. (2008). The predicted toxicity of the hardness effect in the average invertebrate model (dashed line) and the *Daphnia magna* model (dotted line) is also shown. Intrinsic sensitivities are reported in Table S2.4.

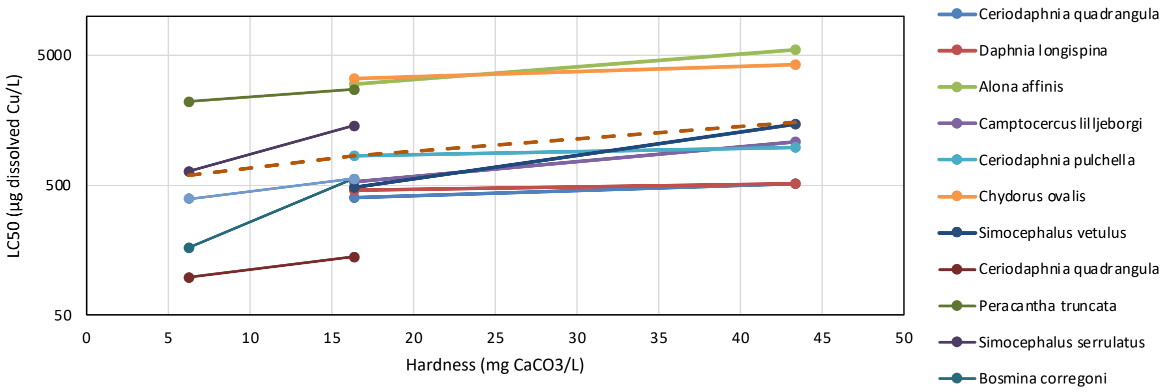

Figure S2. 2 Overview of observed effect (full lines) of hardness on acute Ni toxicity (LC50; µg dissolved Ni/L) for 10 different species of crustaceans. The predicted slope of the hardness effect in the average invertebrate model is also shown (dashed line, calibrated on the toxicity at low hardness for *Simocephalus serratus*).

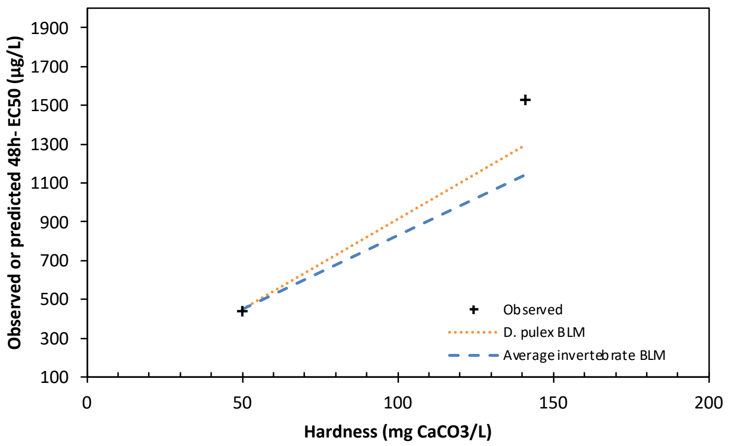

Figure S2. 3 Observed (symbols) versus predicted (lines) 48h-EC50s (µg dissolved Ni/L) for *D. pulex* according to the different bioavailability models in function of hardness. Toxicity data taken from Leonard & Wood (2013). Intrinsic sensitivity was calibrated based on the soft water

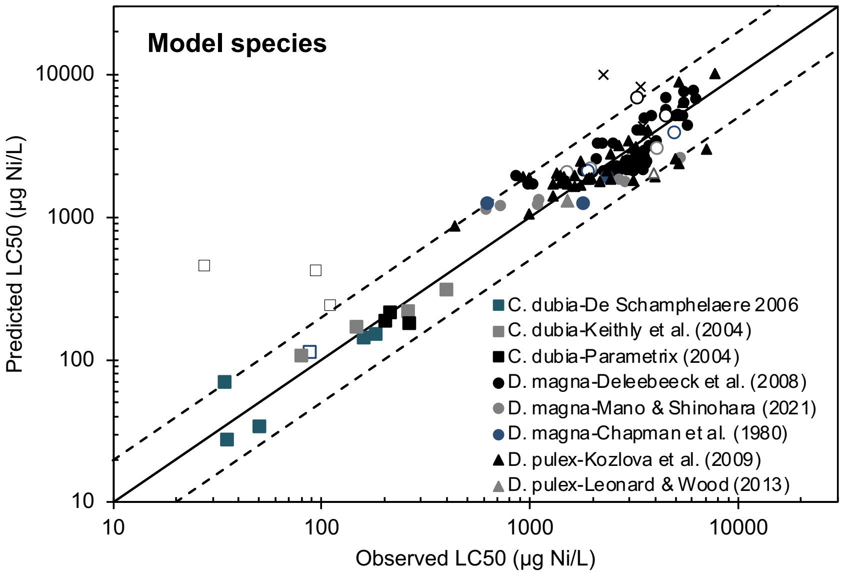

Figure S2. 4 Observed versus predicted LC50s (µg dissolved Ni/L) to both model-species (upper panel) and non-model species (lower panel) predicted with the species-specific bioavailability model (*D. magna* model for *D. magna*; *C. dubia* model for *C. dubia* and *D. pulex* model for *D. pulex)*. The full line represents the perfect prediction line, dashed lines indicate a two-fold prediction error on the observed Ni toxicity. Open symbols represent toxicity data obtained in test solutions with pH>8.0. Crosses indicate that toxicity data has been obtained in test solution with high hardness (>290 mg $CaCO_3$/L). Intrinsic sensitivities have been calibrated for each dataset separately, based on all waters with Ca and Mg<3 mM.

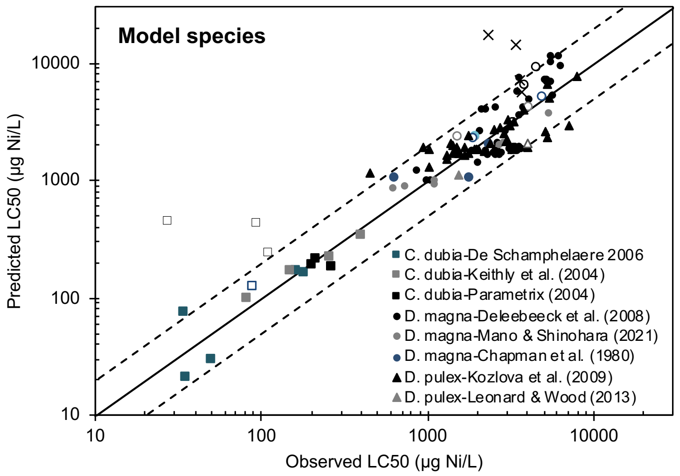

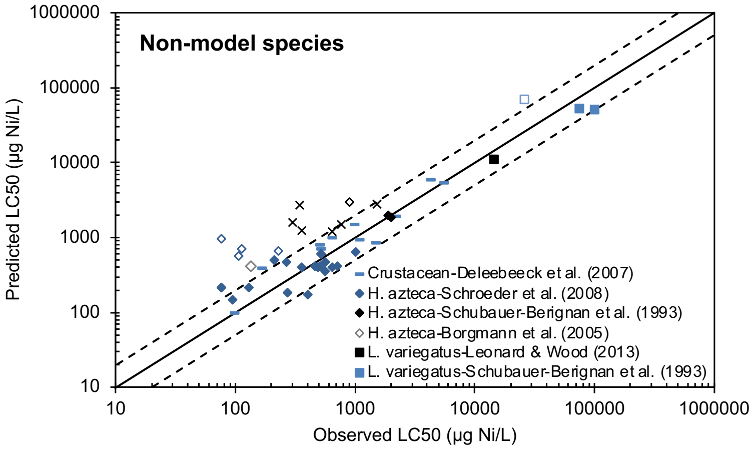

Figure S2. 5 Observed versus predicted LC50s (µg Ni/L) to both model-species (upper panel) and non-model species (lower panel) predicted with the 'basic average invertebrate model'. The full line represents the perfect prediction line, dashed lines indicate a two-fold prediction error on the observed Ni toxicity. Open symbols represent toxicity data obtained in test media with pH>8.0. Crosses indicate that toxicity data has been obtained in test solution with high hardness (>290 mg $CaCO_3$/L). Intrinsic sensitivities have been calibrated for each dataset separately, based on all waters with pH<8.3 and Ca and Mg<3 mM.

Table S2. 7 Overview of residual scores for toxicity modifying factors for the different evaluated bioavailability models

Species	Refer-ence	Toxicity modifying factor (# of samples)	Species-specific model					pH extended average model				
			RS Ca	RS Mg	RS DOC	RS pH	Tot RS	RS Ca	RS Mg	RS DOC	RS pH	Tot RS
Ceriodaphnia dubia	[1]	Natural water (n=6)	0.88	0.87	0.99	0.88	0.91	0.70	0.70	0.94	0.76	0.77
	[2]	Hardness (n=4)	0.59	0.74	-	-	0.66	NA	NA	NA	NA	NA
	[3]	pH (n=6)	-	-	-	0.82	0.82	-	-	-	0.96	0.96
Daphnia magna	[4]	Ca, Mg, Na and pH, Natural water (n=52)	0.90	0.84	0.94	1.00	0.92	0.61	0.99	0.91	0.90	0.85
	[5]	Hardness, pH (n=5)	0.85	0.83	-	0.93	0.87	0.67	0.64	-	0.70	0.67
	[6]	Natural water + pH (n=10)	0.56	0.60	1.00	0.97	0.78	0.75	0.77	0.96	0.92	0.85
Daphnia pulex	[7]	Ca, Mg, K, Na, pH, DOC (n=44)	0.99	0.99	0.94	0.97	0.97	0.91	0.99	0.95	0.93	0.94
	[8]	Hardness (n=2)	0.82	0.73	0.70	0.85	0.78	0.69	0.53	0.48	0.74	0.61
Hyalella azteca	[9]	Hardness (n=2)	NA	NA	NA	NA	NA	0.80	0.84	0.92	0.84	0.85
	[10]	Hardness, pH, alkalinity (n=28)	NA	NA	NA	NA	NA	0.60	0.81	-	0.81	0.74
	[11]	pH (n=3)	NA	NA	NA	NA	NA	-	-	-	0.94	0.94
Lumbriculus variegatus	[8]	Hardness (n=2)	NA	NA	NA	NA	NA	0.95	0.93	0.91	0.96	0.94
	[11]	pH (n=3)	NA	NA	NA	NA	NA	-	-	-	0.71	0.71
10 Crustacean species	[12]	Hardness (n=2)	NA	NA	NA	NA	NA	0.66 (0.25-0.98)	0.57 (0.13-0.97)	-	-	0.61 (0.19-0.97)

Table S2. 8 Performance of the pH extended-average invertebrate model and the average invertebrate model without pH extension for invertebrate species.

**Species**	**Reference[b]**	**Test duration**	**pH range**	**Average invertebrate model without pH extension**				**pH extended average invertebrate model**			
				**r^2 [d]**	**FA[e]**	**Tot RS[f]**	**MPS[g]**	**r^2 [d]**	**FA[e]**	**Tot RS[f]**	**MPS[g]**
Ceriodaphnia dubia	[1]	48h	6.34-8.04	0.81	0.83	0.77	0.80	0.83	0.83	0.77	0.81
	[3]	48h	6.30-8.90	0.00	0.75	0.83	0.53	0.59	1.00	0.96	0.85
Daphnia magna	[4]	48h	5.72-8.13	0.00	0.92	0.84	0.58	0.00	0.94	0.85	0.60
	[5]	48h	7.70-8.30	0.33	1.00	0.99	0.78	0.04	0.80	0.67	0.50
	[6]	48h	6.80-8.20	0.85	1.00	0.94	0.93	0.89	1.00	0.85	0.91
Hyalella azteca	[9]	7d	7.37-8.39	0.00	0.50	0.52	0.34	0.33	1.00	0.85	0.73
	[10]	7d	6.40-8.90	0.50	0.82	0.86	0.73	0.59	0.90	0.86	0.79
	[11]	96h	6.23-8.38	0.95	1.00	0.96	0.97	0.82	1.00	0.94	0.92
Lumbriculus variegatus	[11]	96h	6.57-8.25	0.00	0.67	0.60	0.42	0.37	1.00	0.71	0.69

[a] For model-species the species-specific model indicates that the *C. dubia* model was used for all *C. dubia*-datasets, the *D. magna* model was used for all *D. magna*-datasets *and* the *D. pulex* model was used for all *D. pulex*-datasets.

[b] References: [1]De Schamphelaere et al. 2006; [3] Parametrix 2004; [5] Chapman et al. 1980; [6] Mano & Shinohara 2020; [9] Borgmann et al. 2005; [10] Schroeder et al. 2010; [11] Schubauer-Berignan et al. 1993

[c] Potential toxicity modifying factors (TMF) considered in the dataset. Natural water, indicates that a set of natural waters with different physicochemical conditions has been evaluated. Note that only waters within the applicability ranges of the considered bioavailability model has been considered for calculating performance scores.

[d] r^2 is a metric for the goodness-of-fit of the bioavailability model relative to a null-model in which it is assumed that all variability among toxicity data is attributable to random variation and not to differences in TMF.

[e] Factor agreement (FA) represents the fraction of toxicity data predicted within 2-fold error

[f] Total Residual Score (Tot RS) represents bias in the model predictions relative to the main toxicity modifying factors of the bioavailability model (pH, DOC, Ca, and Mg) considered in the data-set

[g] Model performance score (MPS) represents the average of r^2, FA and Tot RS. MPS is a value between 0 and 1, the higher the MPS the better the performance of the evaluated bioavailability model

h=hours, d= days, DOC= dissolved organic carbon, NA= not applicable

S2.4 Results of the bioavailability modeling for plant species

Schlekat et al. (2010) tested chronic toxicity to *L. minor* in 5 natural waters and a synthetic water. The pH amended S. Platte water was not considered in this analysis, since Schlekat et al. (2010) suggested that toxicity in this water may have been influenced by the presence of pH-sensitive contaminants in the river water other than Ni. Intrinsic sensitivities were calibrated on all other waters (**Error! Reference source not found.**; n=5). For the dataset of Gopalapillai et al. (2013), the intrinsic sensitivity was calibrated on all waters with Ca and Mg ≤3mM (; n=20). For the dataset of Nys et al. (2016), the intrinsic sensitivity was calibrated on the low pH water (pH 8.2) and used to predict Ni toxicity in the high pH water (pH 8.7)

Table S2. 9 **Dataset-specific intrinsic sensitivities for *L. minor* for the different Ni bioavailability models.**

Species	Reference - endpoint	pH extended-average invertebrate model $Q50_{Ni2+}$ (log mol/L)	pH extended algae gBAM $Q50_{Ni2+}$ (log mol/L)
Lemna minor	Schlekat et al. (2010) – growth rate	6.44±0.10	4.93±0.29
	Gopalapillai et al. (2013) – Frond count	6.82±0.30	5.50±0.24
	Nys et al. (2016) – growth rate[a]	6.32	4.65
Lemna aequinoctalis	Peters et al. (2018; n=4) - growth rate	7.11±0.76	5.95±0.65

[a] As the dataset of Nys et al. (2016) only included two datapoints (representing 2 pH levels). The intrinsic sensitivity was calibrated on the low pH water (pH 8.2) and used to predict Ni toxicity in the high pH water (pH 8.7)

The prediction performance for both models for plants is visualized in Figure 5, and prediction statistics are summarized in Table S2.10. For only one of the four datasets for plant species, the pH extended-average invertebrate model performed markedly better compared to the pH extended-algae model, i.e., for the *L. minor* dataset of Schlekat et al. 2010 (MPS 0.93 vs. 0.51), with high scores across the different statistics that are included in the MPS r^2 *(0.92),* FA (1.00), and Tot RS (0.87). None of the bioavailability models resulted in MPS-scores above 0.60 for the other three datasets reporting on Ni toxicity to plant species and all resulted in low scores for the goodness-of-fit statistic (r^2) across datasets (r^2<0.5). Overall, both bioavailability models performed similarly for the *L. minor* datasets of Gopalapillai et al. (2013) and Nys et al. (2016), with MPS-score within 10% of each other. However, for the *L. aequinoctalis* of Peters et al. (2018), the pH extended-algae model performed slightly better (MPS=0.56) compared to the pH extended-average invertebrate model (MPS=0.45). When considering the fraction of toxicity data predicted within two-fold error, the *L. minor* dataset of Gopalapillai et al. (2013) and Nys et al. (2016) were predicted with reasonable accuracy using both bioavailability models (i.e., at least 85% of the toxicity datapoints were predicted within two-fold error), while the *L. aequinoctalis* dataset (Peters et al. 2018) scored also low on this parameter (FA =0.50). It has been earlier reported that bioavailability effects within the *L. aequinoctalis* dataset were difficult to predict with the chronic Ni bioavailability models (Peters et al. 2018). This may suggest that this species does not follow the general bioavailability patterns observed for *L. minor.* However, this observation may also be

the result of differences in exposure duration (96h for *L. aequinoctalis* and 168h for *L. minor*), as bioavailability relationships may shift under prolonged exposure due to acclimation processes (De Schamphelaere & Janssen 2004).

Table S2. 10 Performance of the pH extended-algae mode and the pH extended-average invertebrate model for plant species

Species	Reference[a]	Test duration	Toxicity modifying factor (# of samples) [b]	**pH extended algae model**				pH extended average invertebrate model			
				r^2 [c]	FA[d]	Tot RS[e]	MPS[f]	r^2 [c]	FA[d]	Tot RS[e]	MPS[f]
Lemna minor	[1]	7d	Natural water (n=6)	0.24	0.60	0.69	0.51	0.92	1.00	0.87	0.93
	[2]	7d	Hardness, Na, K (n=25)	0.00	0.86	0.81	0.56	0.00	0.85	0.53	0.52
	[3]	7d	pH (n=2)	0.00	1.00	0.59	0.53	0.00	1.00	0.51	0.50
Lemna aequinoctalis	[4]	96h	Natural water (n=4)	0.46	0.50	0.73	0.56	0.25	0.50	0.60	0.45

[a] [1] Schlekat et al. 2010; [2] Gopalapillai et al. 2013; [3] Nys et al. 2016; [4] Peters et al. 2018

[b] Potential toxicity modifying factors (TMF) considered in the dataset. Natural water, indicates that a set of natural waters with different physicochemical conditions has been evaluated. Note that only waters within the applicability ranges of the considered bioavailability model has been considered for calculating performance scores.

[c] r^2 is a metric for the goodness-of-fit of the bioavailability model relative to a null-model in which it is assumed that all variability among toxicity data is attributable to random variation and not to differences in TMF.

[d] Factor agreement (FA) represents the fraction of toxicity data predicted within 2-fold error

[e] Total Residual Score (Tot RS) represents the bias in the model predictions relative to the main toxicity modifying factors of the bioavailability model (pH, DOC, Ca, and Mg) considered in the data-set

[f] Model performance score (MPS) represents the average of r^2, FA and Tot RS. MPS ranges between 0 and 1, the higher the MPS the better the performance of the evaluated bioavailability model

h=hours, d= days

Table S2. 11 Residual scores for the toxicity modifying factors for plant species for the two evaluated bioavailability models.

Species	Reference	Toxicity modifying factor (# of samples)	pH extended algae model					pH extended average inverteba-rate model				
			RS Ca	RS Mg	RS DOC	RS pH	Tot RS	RS Ca	RS Mg	RS DOC	RS pH	Tot RS
Lemna minor	[1]	Natural water (n=6)	0.63	0.69	0.53	0.93	0.69	1.00	0.73	0.93	0.83	0.87
	[2]	Hardness, Na, K (n=25)	1.00	0.61	-		0.81	0.46	0.96	-	-	0.71
	[3]	pH (n=2)	-	-	-	0.45	0.45	-	-	-	0.51	0.51
Lemna aequinoctalis	[4]	Natural water (n=5)	0.30	0.95	0.77	0.70	0.68	0.12	0.53	0.94	0.80	0.60

[a] [1] Schlekat et al. 2010; [2] Gopalapillai et al. 2013; [3] Nys et al. 2016; [4] Peters et al. 2018

Finally, the prediction bias relative to the toxicity modifying factors (Tot RS) was dependent on the considered dataset and bioavailability model, with relatively-low scores for the pH extended-average invertebrate model across all datasets (Tot RS ≤0.60), except for the dataset of Schlekat et al. (2010). While for the pH extended-algae model, reasonable scores were observed for the *L. minor* dataset of Gopalapillai et al. (2013, Tot RS=0.81) and the *L. aequinoctalis* dataset of Peters et al. (2018; Tot RS=0.73), the dataset of Nys et al. (2016) scored relatively poorly (Tot RS=0.59). Bias in predictions of the pH extended- average invertebrate model were related to Ca (data of Gopalapillai et al. 2013 and Peters et al. 2018), Mg (data of Peters et al. (2018) and pH (data of Nys et al. 2018) (Table S2.11). Bias in the prediction of the pH extended-algae model were related to Ca (data of Peters et al. 2018), Mg (data of Gopalapillai et al. 2013) and pH (data of Nys et al. 2018). The residual variation relative to pH for both bioavailability models observed for the dataset of Nys et al. (2016) is due to the difference in

S_{pH}-parameter in the models at high pH (i.e. 1.01 and 0.906 for the pH extensions of the average invertebrate model and the algae model, respectively) compared to the one previously derived for this specific dataset (0.673, Nys et al. 2016).

Although not fully reflected within the residual scores for the different toxicity modifying factors, the algae model results in clearly biased predictions for the *L. minor* dataset of Gopalapillai et al. (2013), with predictions almost all falling on a horizontal line (green squares in Figure 5 lower panel). This suggest that there are toxicity modifying effects in this *L. minor* dataset that are not accounted for by the algae bioavailability model. Gopalapillai et al. (2013) reported that Ca influenced toxicity of Ni to *L. minor*, which is not considered in the pH extended-algae model (see Table 1). Moreover, the protective effect was dependent on the considered counterion. Protection of Ca on Ni induced growth inhibition was afforded when the media was amended with $CaCl_2$ or $CaSO_4$, but not when $Ca(NO_3)_2$ was used. The variability in effects related to the Ca counter-anion may explain why the residual score analysis did not pick up prediction bias related to Ca for the pH extended-algae model.

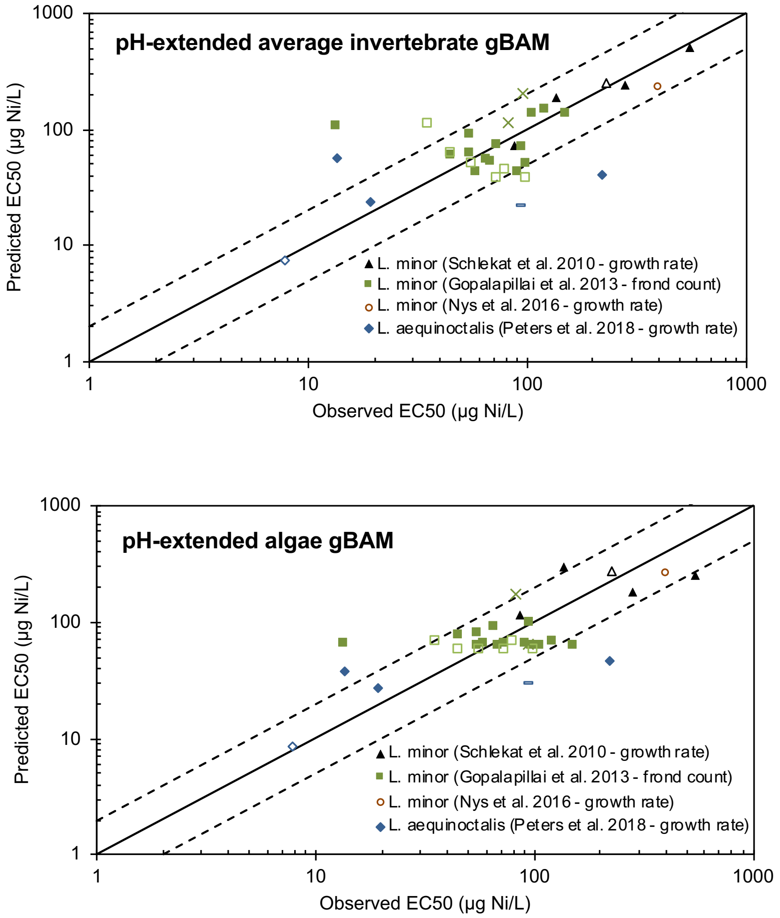

Figure S2. 6 Observed versus predicted EC50s (µg dissolved Ni/L) for plant species predicted with the 'pH extended-average invertebrate model' (upper panel) and the 'pH extended- algae model' (lower panel). The full line represents the perfect prediction line, dashed lines indicate a two-fold prediction error on the observed Ni toxicity. Open symbols represent toxicity data obtained in test solutions with pH>8.0. Crosses indicate that toxicity data has been obtained in test solution with high hardness (>290 mg $CaCO_3$/L), while the striped symbol indicates test solution with Ca<0.02 mM. Intrinsic sensitivities have been calibrated for each dataset separately, based on all waters with Ca between 0.02 and 3 mM and Mg<3mM.

S2.4 References

Borgmann U, Couillard Y, Doyle P. Dixon PG. 2005. Toxicity of sixty-three metals and metalloids to *Hyalella Azteca* at two levels of water hardness. Environmental Toxicology and Chemistry 24(3): 641-652.

Chapman GA, Ota S, et al. 1980. Effects of water hardness on the toxicity of metals to *Daphnia magna*. Corvallis, Oregon, U.S. EPA.

Cheng T, De Schamphelaere K, Lofts S, Janssen C, Allen HE. 2005. Measurement and computation of zinc binding to natural dissolved organic matter in European surface waters. Anal Chim Acta 542:230–239.

De Schamphelaere KAC, Van Laer L, Deleebeeck NME, Muyssen BTA, Degryse F, Smolders E *et al*. 2006. Nickel speciation and ecotoxicity in European natural surface waters: development, refinement and validation of bioavailability models. Report prepared for the Nickel Producers Environmental Research Association (NiPERA), Durham, NC, USA, Gent University, Laboratory of Environmental Toxicology and Aquatic Ecology, Gent, Belgium.

Deleebeeck NME, De Schamphelaere KAC, Heijerick DG, Bossuyt BTA, Janssen CR. 2008. The acute toxicity of nickel to Daphnia magna: Predictive capacity of bioavailability models in artificial and natural waters. Ecotoxicology and environmental safety 70(1): 67-78.

Deleebeeck NME, Muyssen BTA, De Laender F, Janssen CR, De Schamphelaere KAC. 2007. Comparison of nickel toxicity to cladocerans in soft versus hard surface waters. Aquat Toxicol 84:223–35.

Deleebeeck NME, De Schamphelaere KAC, Janssen CR. 2009. Effects of Mg2+ and H+ on the toxicity of Ni2+ to the unicellular green alga Pseudokirchneriella subcapitata:model development and validation with surface waters. Sci Tot Environ 2009a;407: 1901–14.

Gopalapillai, Y., Beverley H., Vigneault, B. 2013. Effect of major cations (Ca2+, Mg2+, Na+, K+) and anions (SO42-, Cl-, NO3-) on Ni accumulation and toxicity in aquatic plant (Lemna minor L.): Implications for Ni risk assessement, Environmental Toxicology and Chemistry, vol. 32, iss. 4, pp. 810-821.

Keithly J, Brooker JA, DeForest DK, Wu BK, Brix KV. 2004. Acute and chronic toxicity of nickel to a cladoceran (*Ceriodaphnia dubia*) and an amphipod (*Hyalella azteca*). Environmental Toxicology and Chemistry 23: 691-696.

Kozlova T, Wood CM, McGeer JC. The effect of water chelistry on the acute toxicity of nickel to the cladoceran *Daphnia pulex* and the development of a biotic ligand model. Aquatic Toxicology 91(3): 221-228.

Leonard EM, Wood CM 2013. Acute toxicity, critical body residues, Michealis-Menten analysis of bioaccumulation, and ionoregulatory disturbance in response to waterborne nickel in four invertebrates: *Chironomus, riparius, Lymnaea stagnalis, Lumbriculus variegatus* and *Daphnia pulex.* Comparative Biochemistry and Physiology C-Toxicology & Pharmacology 158 (1): 10-21.

Mano H, Shinohara N. 2020. Acute toxicity of nickel to *Daphnia magna*: Validation of Bioavailability models in Japanese Rivers. Water, Air, & Soil Pollution 231: 459.

Nys C, Janssen CR, Van Sprang P, De Schamphelaere KAC. 2016. The effect of pH on chronic aquatic Ni toxicity is dependent on the pH itself: extending the chronic Ni bioavailability models. Environmental Toxicology and Chemistry 35: 1097-1106.

Parametrix. 2004. Níckel toxicity to *Ceriodaphnia dubia*. August 2004 Version. Report prepared for Níkel Producers Environmental Research Association (NiPERA), Durham, NC, USA. Parametrix, Inc., Albany, OR, USA.

Peters A, Merrington G, Schlekat C, de Schamphelaere K, Stauber J, Batley G, Harford A, van Dam R, Pease C, Mooney T, Warne M, Hickey C, Glazebrook P, Chapman J, Smith R, Krassoi R. 2018. Validation of the nickel biotic ligand model for locally relevant species in Australian freshwaters. Environmental Toxicology and Chemistry 37:2566-2574.

Ritchie JD, Perdue EM. 2003. Proton-binding study of standard and reference fulvic acids, humic acids, and natural organic matter. Geochimica et Cosmochimica Acta 67 (1): 85-96.

Schlekat CE, Van Genderen E, De Schamphelaere KAC, Antunes PMC, Rogevich E, Stubblefield WA. 2010. Cross-species extrapolation of chronic nickel Biotic Ligand Models. Science of the Total Environment 408**:** 6148-6157.

Schubauer-Berigan MK, Dierkes JR, Monson PD, Ankley GT, 1993. pH-dependent toxicity of Cd, Cu, Ni, Pb and Zn to Ceriodaphniadubia, Pimephales promelas, Hyalella Azteca and Lumbriculus variegates. Environ. Toxicol. Chem. 12: 1261–1266.

Schroeder JE, Borgmann U, Dixon DG. 2010. Evaluation of the biotic ligand model to predict long-term toxicity of nickel to *Hyalella azteca. Environmental Toxicology and Chemistry 29*: 2498-2504.

Tipping E. 1998. Humic ion-binding model VI: An improved description of the interactions of protons and metal ions with humic substances. Aq.Geochem. 4: 3-48.

Van Laer L, Smolders E, Degryse F, Janssen C, De Schamphelaere KAC. 2006. Speciation of nickel in surface waters measured with the Donnan membrane technique. Analytica Chimica Acta 578: 195-202.

S3: Overview of detailed physico-chemistry of ecoregion scenarios used for calculating local $HC5_{L(E)C50}$ and fitted species sensitivity distributions

Table S3. 1 Overview of detailed physico-chemistry of ecoregion scenarios used for bioavailability modeling

Sample Name	Temp (°C)	pH	DOC (mg/L)	Ca (mg/L)	Mg (mg/L)	Na (mg/L)	K (mg/L)	Cl (mg/L)	SO_4 (mg/L)	CO_3 (M)
Lake Monate	15.0	7.7	2.5	13.6	3.5	2.3	0.8	3.2	18.8	1.06E-03
Rhine	15.0	7.8	2.8	68.9	10.9	36.8	5.7	81.5	51.9	2.46E-03
Otter	15.0	8.1	3.2	46.9	11.6	14.2	5.4	23.5	34.0	2.35E-03
Teme	15.0	7.6	8.0	50.1	8.4	12.9	3.7	22.3	22.7	2.50E-03
Swedish lake	15.0	6.7	3.8	8.7	1.5	7.7	0.9	11.6	10.7	4.03E-04
Ebro	15.0	8.2	3.7	72.9	22.1	5.3	1.1	5.6	6.6	7.20E-04
Ditches adjusted DOC + H	15.0	6.9	12.0	60.1	26.7	59.8	8.4	113.4	76.0	6.90E-03

Lake Monate

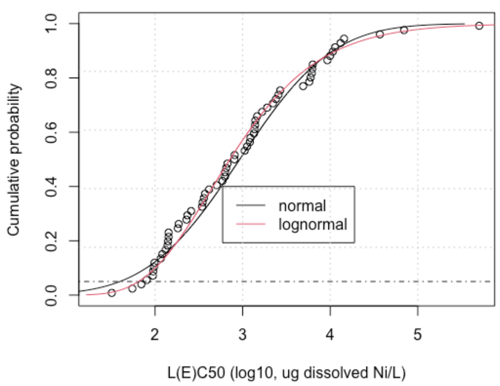

Rhine

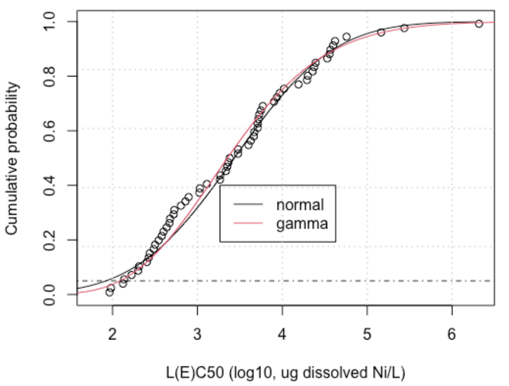

Otter

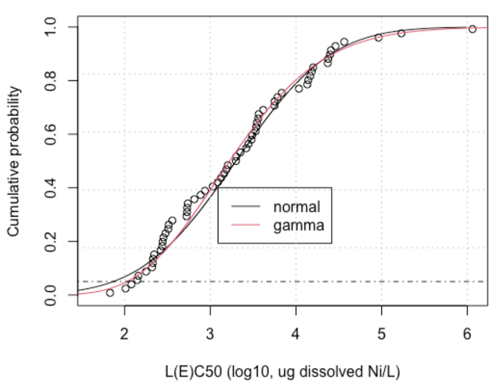

Teme

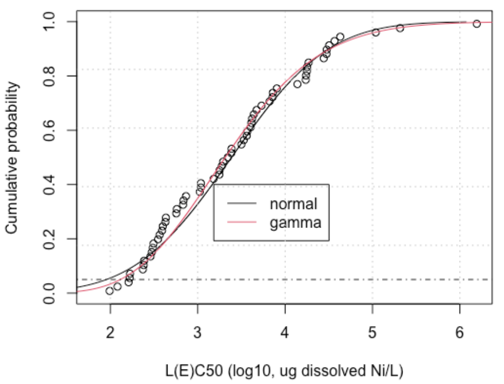

Swedish Lake

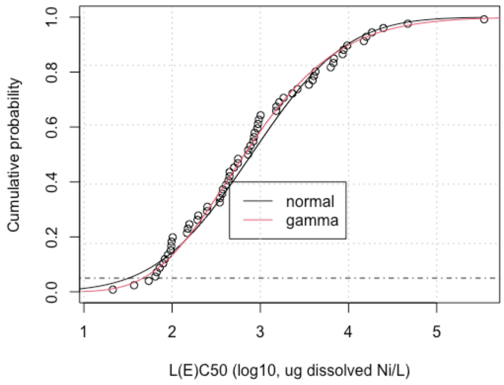

Ebro

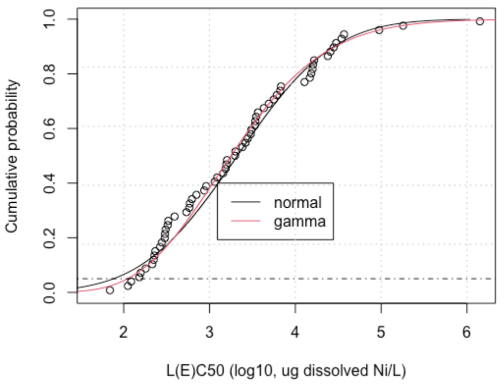

Ditches

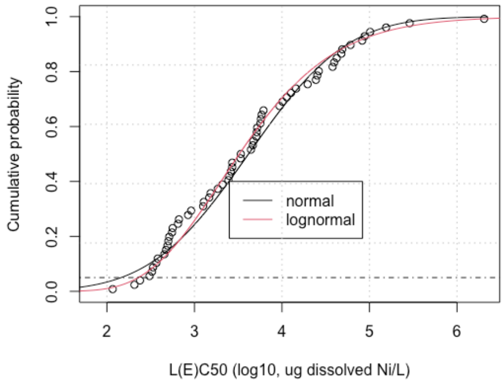

Figure S3. 1 Species sensitivity distribution of normalised mean LC50/EC50 values (symbols; each symbol represents one species) from the acute Ni freshwater toxicity database for the ecoregion scenarios. The black line represents the fit of the normal distribution; the red line represents the fit of the best-fitting distribution (log-normal distribution for all scenarios, except Rhine). Distributions have been fitted to species-mean bioavailability-normalized log_{10} transformed LC50/EC50 values. Best-fitting distribution has been selected based on the Anderson-Darling statistic. The horizontal dashed/dotted line represents the position of 5% potentially affected species.

Table S3. 2 **Normalised species-average L(E)C50 for most sensitive endpoint (in µg dissolved Ni/L) in the 7 'ecoregion'-scenarios**

Species	Model used for normalisation	Most sensitive endpoint	Lake Monate	Rhine	Otter	Teme	Swedish lake within boundary	Ebro	Ditches adjusted DOC + H
Bufo terrestris	Fish model	mortality	1668	5140	3635	4719	2310	4293	12684
Duttaphrynus melanostictus	Fish model	mortality	6326	19967	13916	17732	8787	16545	48800
Gastrophryne carolinensis	Fish model	malformation	372	1066	773	1057	503	912	2701
Xenopus laevis	Fish model	malformation	258	722	528	734	346	624	1846
Ambloplites rupestris	Fish model	mortality	1904	5884	4154	5377	2635	4900	14484
Catla catla	Fish model	mortality	4909	15491	10816	13833	6824	12782	37900
Danio rerio	Fish model	mortality	6167	19520	13568	17322	8566	16074	47646
Lepomis macrochirus	Fish model	mortality	11372	36060	24993	31872	15904	29824	88039
Oncorhynchus mykiss	Fish model	mortality	13129	41756	28896	36795	18451	34544	101780
Pimephales promelas	Fish model	mortality	2569	7999	5623	7248	3557	6649	19649
Poecilia reticulata	Fish model	mortality	10704	33826	23498	29924	14934	27937	82649
Rasbora sumatrana	Fish model	mortality	629	1853	1337	1779	862	1569	4649
Alathyria profuga	Invertebrate model	Mortality	143	475	328	432	98	331	563
Alona affinis	Invertebrate model	Immobilization	6226	24663	15884	18552	4225	15886	26472
Bosmina coregoni	Invertebrate model	Immobilization	810	3025	2009	2437	559	2003	3386
Branchinecta lindahli	Invertebrate model	Mortality	1397	5331	3505	4184	962	3495	5863
Branchinecta lynchi	Invertebrate model	Mortality	1209	4609	3031	3637	832	3028	5085
Camptocercus lilljeborgi	Invertebrate model	Immobilization	1226	4677	3079	3689	847	3066	5165
Ceriodaphnia dubia	Invertebrate model	mortality	80	253	178	243	54	180	306
Ceriodaphnia pulchella	Invertebrate model	Immobilization	1465	5591	3670	4383	1007	3666	6153
Ceriodaphnia quadrangula	Invertebrate model	Immobilization	506	1842	1230	1517	349	1229	2092
Chironomus dilutus	Invertebrate model	mortality	69674	273324	169967	206686	47033	180054	287860
Chironomus javanus	Invertebrate model	mortality	9953	39634	25567	29719	6736	25581	42261
Chydorus ovalis	Invertebrate model	Immobilization	5767	22763	14727	17219	3917	14757	24414
Cucumerunio novaehollandia	Invertebrate model	Mortality	95	305	214	288	65	215	367
Daphnia longispina	Invertebrate model	Immobilization	805	3002	1997	2415	555	1984	3359
Daphnia magna	Invertebrate model	mortality & immobolization	2225	8617	5635	6646	1520	5610	9383
Daphnia pulex	Invertebrate model	mortality	2686	10458	6817	8035	1834	6800	11347
Daphnia pulicaria	Invertebrate model	mortality	1346	5140	3386	4047	931	3371	5666
Gammarus pulex	Invertebrate model	mortality	502886	2076341	1149147	1560519	344988	1415084	2043402
Hyalella azteca	Invertebrate model	7d mortality	228	787	536	688	157	538	917
Hyridella australis	Invertebrate model	Mortality	99	317	221	298	67	224	380
Hyridella depressa	Invertebrate model	Mortality	116	380	264	352	79	265	453
Hyridella drapeta	Invertebrate model	Mortality	121	398	275	366	83	278	474
Lampsilis abrupta	Invertebrate model	Immobility & Mortality	1056	3988	2628	3161	726	2627	4424
Lampsilis cardium	Invertebrate model	Immobility & Mortality	651	2400	1601	1952	449	1598	2701
Lumbriculus variegatus	Invertebrate model	mortality	9285	36953	23843	27670	6280	23886	39439
Lymnaea stagnalis	Invertebrate model	mortality	361	1292	867	1087	249	868	1474
Melanoides tuberculata	Invertebrate model	mortality	14386	57058	36565	42752	9671	36908	60721
Neocloeon triangulifer	Invertebrate model	Mortality	132	434	301	398	90	303	515
Peracantha truncata	Invertebrate model	Immobilization	5976	23657	15306	17834	4057	15322	25314
Physa gyrina	Invertebrate model	Mortality	630	2321	1541	1887	434	1540	2607
Simocephalus serrulatus	Invertebrate model	Immobilization	2401	9325	6092	7186	1648	6073	10151

Simocephalus vetulus	Invertebrate model	Immobilization	1363	5219	3434	4100	939	3419	5745
Stenocypris major	Invertebrate model	mortality	36911	145875	92007	108962	24807	94886	154739
Thamnocephalus platyurus	Invertebrate model	Mortality	590	2161	1445	1768	407	1443	2445
Utterbackia imbecilis	Invertebrate model	Immobility & Mortality	1124	4248	2794	3362	772	2789	4702
Velesunio ambiguus	Invertebrate model	Mortality	141	468	324	426	96	325	555
Ankistrodesmus falcatus	Algae model	growth rate	414	638	652	680	447	704	1529
Chlamydomonas sp.	Algae model	growth rate	143	205	215	241	151	233	503
Chlorella sp. - scandinavian strain	Algae model	growth rate	1388	2241	2247	2211	1513	2416	5204
Chlorella sp. - Australian strain	Algae model	growth rate	671	1054	1069	1088	728	1151	2499
Chlorella sp. - Papua New Guinea strain	Algae model	growth rate	70	96	103	121	72	112	239
Coelastrum microporum	Algae model	growth rate	344	525	538	568	370	581	1257
Desmodesmus sp.	Algae model	growth rate	349	533	546	576	377	590	1278
Desmodesmus spinosus	Algae model	growth rate	182	267	278	306	194	301	649
Pediastrum duplex	Algae model	growth rate	94	133	140	162	99	152	328
Pseudokirchneriella sp.	Algae model	growth rate	139	200	210	236	147	228	491
Pseudokirchneriella subcapitata	Algae model	yield	97	137	145	167	101	158	337
Scenedesmus accuminatus	Algae model	growth rate	186	274	284	312	198	308	665
Spermatosopsis exsultans	Algae model	growth rate	234	349	361	391	251	391	846
Lemna aequinoctialis	Invertebrate model	growth rate	32	92	68	98	21	69	116
Lemna minor	Invertebrate model	Frond count	57	174	124	172	38	126	212

Table S3. 3 Comparison of bioavailability-normalized site-specific acute environmental thresholds[a] calculated for 7 European freshwater scenarios (i.e. the 'ecoregions') for both the best-fitting distribution and the normal distribution[b]

Ecoregion	pH	DOC (mg/L)	Hardness (mg $CaCO_3$/L)	Best-fit distribution		Normal distribution[d]	
				$HC5_{L(E)C50}$ [c] (µg Ni/L)	AD-statistic; p-value Best-fit distribution	$HC5_{L(E)C50}$ [c] (µg Ni/L)	AD-statistic; p-value
Lake Monate (It)	7.7	2.5	48	66.3 (46.4-96.8)	A=0.34; p=0.42 Log-normal	38.2 (19.1-67.2)	A=0.60; p=0.12
Rhine (Nl)	7.8	2.8	217	137 (73.1-259)	A=0.46; p=0.76 Gamma	80.1 (37.3-149)	A=0.56; p=0.14
Otter (UK)	8.1	3.2	165	120 (66.8-215)	A=0.44; p=0.72 Gamma	71.0 (34.4-128)	A=0.59; p=0.12
Teme (UK)	7.6	8.0	160	148 (80.9-267)	A=0.46; p=0.73 Gamma	86.6 (42.0-156)	A=0.63; p=0.09
Swedish Lake (SE)	6.7	3.8	28	52.6 (30.6-90.2)	A=0.25; p=0.75 Gamma	31.0 (15.3-55.0)	A=0.52; p=0.18
Ebro (Sp)	8.2	3.7	273	126 (68.9-224)	A=0.42; p=070 Gamma	72.9 (35.1-132)	A=0.59; p=0.12
Ditches (Nl)	6.9	12.0	260	233 (156-363)	A=0.38; p=0.35 Log-normal	139 (66.7-254)	A=0.58; p=0.12

[a] Represented by the $HC5_{L(E)C50,}$the acute 5% hazardous concentration (i.e. concentration that results in at least 50% effect for 5% of the species)

[b] All distributions have been fitted to bioavailability-normalized log_{10} transformed species-mean L(E)C50-values.

[c] The median HC5 (i.e. HC5-50) is reported. The 90% confidence interval on the HC5-50 is reported between brackets (HC5-5 to HC5-95).

[d] Normal distribution HC5-values have been calculated using the approach of Aldenberg & Jaworska (2002)

DOC= dissolved organic carbon

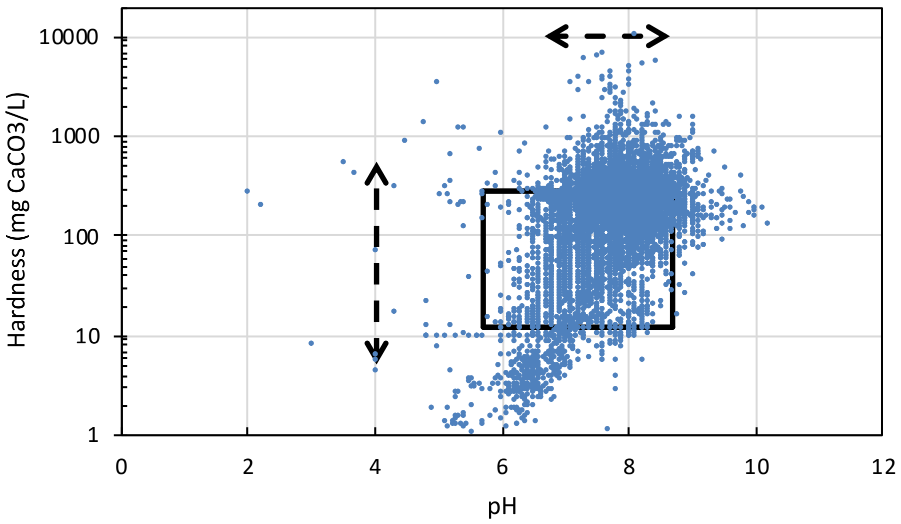

Figure S3.2 Overview of pH and hardness conditions in European waters reported in the EU Physicochemical Database (https://mera-rapps.shinyapps.io/EU_PhysChem_DB_Tool/, consulted on 14/06/2024). Lines indicate the applicability ranges of the acute environmental threshold derivation approach (pH 5.7-8.7 and hardness 12-290 mg $CaCO_3$/)L. Arrows indicate the 95th percentile in pH (horizontal; pH 6.7-8.6) and hardness (vertical; hardness 5.1-560.1 mg CaCO3/L) in the EU physicochemical database.

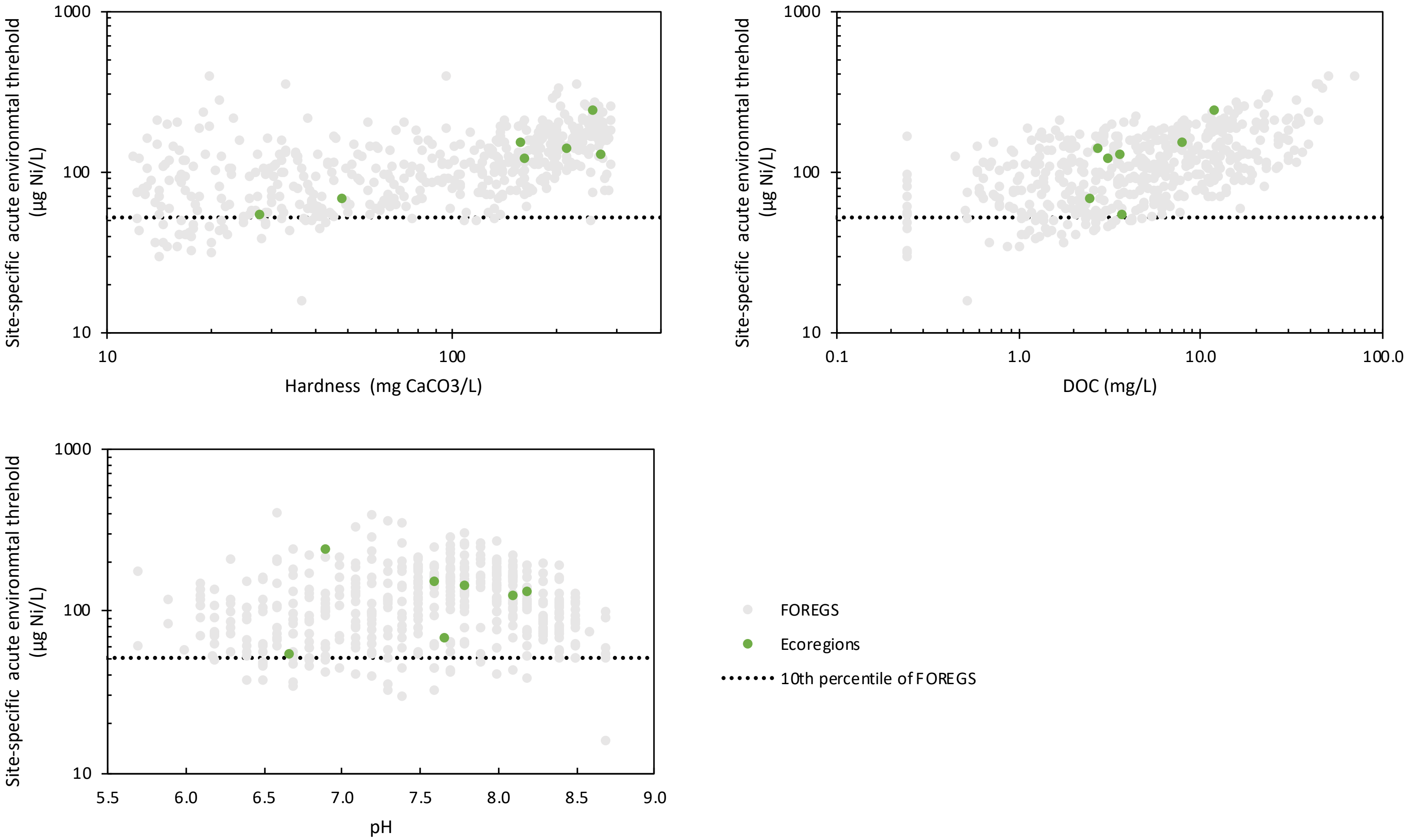

Figure S3. 3 Relationship between site-specific acute environmental threshold ($HC5_{L(E)C50}$, expressed as µg dissolved Ni/L) and the main toxicity modifying factors: hardness (upper left panel), DOC (upper right panel) and pH (lower panel). Both surface waters of the FOREGS database (grey symbols) and those of the ecoregions (green symbols) are indicated. The dashed line represents the 10th percentile of site-specific $HC5_{L(E)C50}$ in the FOREGS database.
