# Supplementary material for: Development of a bioavailability-based acute effects assessment method for nickel
Source: Environ Toxicol Chem. 2025 Jan 6;44(3):841–55. doi: 10.1093/etojnl/vgae071 (PMC11864204; doi:10.1093/etojnl/vgae071)
Supplement: vgae071_Supplementary_Data [file vgae071_supplementary_data.zip › vgae071_Supplementary_Data/S4_Acute Ni bioavailability tool.zip!/Acute Ni bioavailability tool/S4_manual_acute Ni bioavailability tool.docx]

Supplemental information 4: Manual of the ‘Acute Ni bioavailability tool’

Acute Ni toxicity data is normalized to the physicochemical properties of up to 10 target waters in the ‘Acute Ni bioavailability tool’, which is provided as supplemental information to the manuscript ‘*Development of a bioavailability-based acute effects assessment method for Ni* ‘. The outline of the Ni normalisation procedure is vizualized in Figure 1, this figures also includes the corresponding excel-sheets were the different steps in the normalisation procedure have been integrated.

Figure 1. Overview of the acute Ni bioavailability normalisation approach. The acute Ni bioavailability normalisation approach combines the acute Ni toxicity dataset with the acute Ni bioavailability model set and the species-sensitivity distribution (SSD)-approach. The bioavailability normalisation procedure starts with translating the acute Ni toxicity database, with Ni toxicity expressed as dissolved or total concentration, to free ion activities in WHAM VI. The free Ni^2+^ toxicity and Mg^2+^ and Ca^2+^ activity resulting from these calculations are used to calculate an intrinsic sensitivity (Q50) for each toxicity-data entry separately using the appropriate taxon-specific bioavailability model selected from the acute Ni bioavailability model set (i.e., use of bioavailability models in Direction 1, see S3). After species-averaging and most sensitive endpoint selection based on the intrinsic sensitivity values, the species-specific average intrinsic sensitivity is used for predicting Ni^2+^ toxicity in a target water using the taxon-specific bioavailability model selected from the bioavailability model set and WHAM VI-calculated free Ca^2+^ and Mg^2+^ activities and the pH of the target water (i.e., use of bioavailability models in Direction 2, see S3). This results in a list of normalised Ni^2+^ toxicities: one for each species per target water, expressed at the free ion activity level. These Ni^2+^ toxicities are then translated back to dissolved Ni concentrations using WHAM VI and the target water-specific physico-chemistry. Finally, a site-specific acute environmental threshold, the HC5_L(E)C50_ (i.e. concentration resulting in 50% effect for 5% of the species in the SSD) is calculated.

1. Assumptions and adapted databases

The normalization of the acute Ni toxicity dataset can be conducted in **Excel** via the **‘Acute Ni bioavailability tool’**. This Excel file is used together with the speciation software **WHAM VI** (Tipping et al. 1998). The default stability constants for inorganic complexes in WHAM should be adapted to those of NIST (*National Institute of Standards and Technology,* Table S6.1). The adapted master database is enclosed in the supplementary WHAM files as ‘***ni_rar_2008.dbm’***. Furthermore, also some of the default fulvic acid binding constants should be adapted to the values of De Schamphelaere et al. (2006) (Table S6.2). The adapted phase database for fulvic acid (FA) is enclosed in the normalization files (‘***FA KNi1,75.sdb’****)*. Note that these same files are also the master and phase database used in the chronic Ni normalisations (see Nys et al. 2016; Peters et al. 2023). These files have to be saved in the ‘Databases’ directory of the ‘WHAM6’ folder before starting the normalization procedure. For DOM from natural sources (e.g. tap water, surface water), it is assumed in the normalization procedure that 40% of the DOM is reactive and behaves as isolated FA, with the remaining 60% of the DOM content not being reactive. Accordingly, the measured DOC content (mg C/L) for natural sources has to be multiplied by a factor of 0.8 to obtain the amount of FA (mg FA/L) to be used as the modeling input. Furthermore, it is assumed that activities of the metal cations Fe^3+^ and Al^3+^ **-** both competing with Ni^2+^ for binding sites on FA – are controlled by their colloidal Fe(OH)_3_ and Al(OH)_3_ precipitates (De Schamphelaere et al. 2006).

Table S6.1: Default and adapted stability constants for inorganic complexes. Stability constants were adapted to those of NIST.

| Element | Parameter | Default stability constant in WHAM VI (log K) | Adapted stability constant (log K) |
| --- | --- | --- | --- |
| Ni | K = [NiCO_3_]/{[Ni^2+^].[CO_3_^2-^]} | 5.78 | 4.57 |
|  | K = [NiHCO_3_^+^]/{[Ni^2+^].[H^+^].[CO_3_^2-^]} | 13.41 | 12.42 |
| Cu | K = [CuCO_3_]/{[Cu^2+^].[CO_3_^2-^]} | 6.75 | 6.77 |
|  | K = [CuHCO_3_^+^]/{[Cu^2+^].[H^+^].[CO_3_^2-^]} | 14.62 | 12.129 |
| Zn | K = [ZnCO_3_]/{[Zn^2+^].[CO_3_^2-^]} | 4.76 | 4.76 |
|  | K = [ZnHCO_3_^+^]/{[Zn^2+^].[H^+^].[CO_3_^2-^]} | 13.12 | 11.829 |
| MOPS | K=[H(MOPS)]/{[H^+^].[MOPS^-^]} | Not in database | 7.2 |

TableS6.2: Default stability constants of WHAM 6.1 and adapted stability constants of Ni for fulvic acid.

| Element | Parameter | Default value | Adapted value |
| --- | --- | --- | --- |
| Ni/NiOH | LogKMA(FA) | 1.4 | 1.75 |
| Zn/ZnOH | LogKMA(FA) | 1.6 | 1.8 |

2. Content of the ‘extended chronic Ni bioavailability normalization tool’

The ‘extended chronic Ni bioavailability normalization tool’ contains different sheets, in which the toxicity data is normalized step by step to the physicochemical properties of the target water *t*. The content of each sheet is explained in the ‘guide to the file’, but will also be explained below.

The first sheet **‘1.a Acute Ni dataset’** contains the acute Ni database. The second sheet ‘1.b Excluded data’ contains an overview of non-retained data. In sheet **‘2. WHAM input acute dataset’**, the data input for the calculation of the speciation of the Ni toxicity data in a WHAM format is shown. The speciation calculations were already run in WHAM and the results are shown in the sheet **‘3. Intrinsic sensitivity calc’.** In this sheet, the resulting free Ni^2+^ toxicity and Mg^2+^ and Ca^2+^ activity are used to calculate an intrinsic sensitivity (Q50) for each toxicity-data entry separately using the appropriate taxon-specific bioavailability model selected from the acute Ni bioavailability model set (i.e., use of bioavailability models in Direction 1). Within this sheet, species-averaging and most sensitive endpoint selection is also performed, resulting in one species-mean intrinsic sensitivity value per species. In sheet four (**‘4. WHAM input target water’**) the WHAM input for the target waters can be summarized. Default Al^2+^ and Fe^2+^ activities are again calculated based on the maximum solubility. With these input data the speciation in the target water can be calculated in WHAM. The calculated activities of Ca^2+^ and Mg^2+^ in the target water will be used further on to normalize the data.

Normalisation of the species-mean intrinsic sensitivity is performed in sheet ‘**5. Bioav. Normalisation’,** all calculations are automatically performed. The output is a set of predicted geomean-species L(E)C50, expressed as free Ni2+ activities, one for each species in the toxicity database and per target water. The predicted L(E)C50, expressed as Ni^2+^ activities are used to calculate dissolved Ni concentrations in WHAM with the input file in sheet ‘**6. WHAM input_Ni diss calc’**. The output of the WHAM 6-speciation calculations can be pasted in sheet ‘**7. WHAM output_Ni diss’**. In sheet ‘**8. Norm. species LC50 and HC5’**, all geomean species L(E)C50, expressed as dissolved concentrations, are summarized for each target water. This output can subsequently be used to perform the best-fit distribution analysis. A normal HC5-50 is also automatically calculated using the Aldenberg and Jaworska (2000).

3. How to use the chronic Ni bioavailability normalization tool:

Throughout the ‘extended chronic Ni bioavailability normalization tool’ sheets and cells that have to be filled in by the user are marked blue. The normalization procedure starts with filling in the physicochemical properties of the target waters, up to 10 target waters can be run simultaneosly. This is done in row 5-14 of sheet **‘4. WHAM input target water’**. pH and concentrations of DOC, Na, Mg, K, Ca, Cl, SO_4_, CO_3_ are needed to adequately calculate the HC5. The Excel file will calculate default Al^2+^ and Fe^2+^ activities based on the pH, temperature and the maximum solubility and summarizes the WHAM input in row 22-31. With the physicochemical properties of the target water the Mg^2+^ and Ca^2+^ activities can be calculated with WHAM VI. To do this open in WHAM a new input file. First, the default phase database has to be changed to the adapted phase database. This can be done by right clicking on the input file and selecting ‘Phase configuration’: select Fulvic Acid and press edit, select **‘*FA KNi1,75’.sdb’*** below database***.*** When changing the FA phase database, WHAM will ask you to restart the program, click ‘Yes’. Open the input file ***‘2024_Nitargetwater.wdt’***. Second, the default master database has to be changed. The master database can be changed by right clicking on the input file and selecting ‘Options’. Now you can select the ***‘ni_RAR_2008’ database*** ***.*** Next, paste the target water chemistry summarized in sheet 4 into the input file. Click on ‘Run_Speciation Model’ to calculate the speciation, select under the sheet ‘Components’ of the output menu the Free ion activity of Mg and Ca and click on OK. Paste these values in cell rows 37-46, respectively. With these inputs, the Excel file will now automatically normalize the acute Nu toxicity dataset to the physicochemical properties of the target waters.

Fill in physicochemical properties of target waters

Paste water chemistry in WHAM

Fill in WHAM calculated Mg2+ and Ca2+ activities

In sheet **‘5. Bioav. normalisation’, intrinsic sensitivities are normalized to conditions of the target water and summarized is** as Ni^2+^ activities for all target waters. The WHAM input data to calculate the dissolved Ni concentrations (mol/L) from the normalized Ni^2+^ activities and the physicochemical properties of the target water are listed in sheet ‘7. **WHAM input_Ni diss calc’**). Paste these data in WHAM in the input file ***‘2024_Nidisscalc’***. Let the speciation model run and select ‘Total Ni Component’ in the Component sheet of the output menu. Paste the dissolved Ni concentrations in column D of sheet (**‘7. WHAM output_Ni diss’**). The output of the acute Ni normalisation is summarized in sheet ‘8. Norm species LC50 and HC5’. This sheet summarized the HC5 and species-mean L€C50 for the different target waters. Please note that the best-fit distribution fitting itself is not programmed into the excel file, but can be done in R as described in the main paper.

Summary of the species mean normalized L(E)C50, which are the input for best fit distribution analysis

HC5 following the normal distribution is automatically calculated

Paste output of the best fit distribution fitting – the best-fit calculations are performed in R
